# Enhancing Biomarker Detection and Imaging Performance of Smartphone Fluorescence Microscopy Devices

**DOI:** 10.3390/bios15070403

**Published:** 2025-06-21

**Authors:** Muhammad Ahsan Sami, Muhammad Nabeel Tahir, Umer Hassan

**Affiliations:** 1 Department of Electrical and Computer Engineering, School of Engineering, Rutgers, The State University of New Jersey, New Brunswick, NJ 08901, USA; ahsan.sami@rutgers.edu (M.A.S.); nabeel.tahir@rutgers.edu (M.N.T.); 2 Global Health Institute, Rutgers, The State University of New Jersey, New Brunswick, NJ 08901, USA

**Keywords:** smartphone fluorescence microscope (SFM), micro/nanoparticles, leukocytes, image quality enhancement, imaging filters

## Abstract

Fluorescence microscopy enabled by smartphone-coupled 3D instruments has shown utility in different biomedical applications ranging from diagnostics to biomanufacturing. Recently, we have designed and developed these devices and have demonstrated their utility in micro-nano particle sensing and leukocyte imaging. Here, we present a novel application for enhancing the imaging performance of smartphone fluorescence microscopes (SFM) and reducing their operational complexity. Computational noise correction is employed using 3D Averaging and 3D Gaussian filters of different kernel sizes (3 × 3 × 3, 7 × 7 × 7, 11 × 11 × 11, 15 × 15 × 15, and 21 × 21 × 21) and various standard deviations σ (for Gaussian only). Fluorescent beads of different sizes (8.3, 2, 1, 0.8 µm) were imaged using a custom-designed SFM. The application of the computational filters significantly enhanced the signal quality of particle detection in the captured fluorescent images. Amongst the Averaging filters, a kernel size of 21 × 21 × 21 produced the best results for all bead sizes, and similarly, amongst Gaussian filters, σ equal to 5 and a kernel size equal to 21 × 21 × 21 produced the best results. This visual improvement was then quantified by calculating the signal-difference-to-noise ratio (SDNR) and contrast-to-noise ratio (CNR) of filtered and unfiltered original images using a custom-developed quality assessment algorithm (AQAFI). Lastly, noise correction using Averaging and Gaussian filters with the previously identified optimal parameters was applied to images of fluorescently tagged human peripheral blood leukocytes captured using an SFM under various conditions. The ubiquitous nature and simplistic application of these filters enable their utility with a range of existing fluorescence microscope designs, thus allowing us to enhance their imaging capabilities.

## 1. Introduction

Fluorescence microscopes are perhaps one of the fundamental analytical instruments and are thus present in almost all modern-day laboratories working on biology, chemistry, and material sciences [1,2,3,4,5,6]. Although the current fluorescence microscopes are state-of-the-art instruments, expensive procurement, lack of portability, and the requirement of lab-trained users limit their use in low-resource and point-of-care (POC) settings [7]. Smartphone fluorescence microscopes (SFMs) are being touted as a potential solution to the limitations faced by regular benchtop fluorescence microscopes [8]. Modern smartphones are equipped with a lot of computing power, which allows for local processing of the captured fluorescence images, thus allowing for greater use of SFMs for POC applications, healthcare applications, and environmental monitoring [9,10,11,12].

Multiple studies using SFM for different biomedical applications have been reported by researchers in the past [13,14,15,16,17,18,19,20,21,22,23,24,25,26]. Furthermore, the working of a smartphone-based fluorescence microscope in conjunction with a microfluidic biosensor for the quantitative detection of *Salmonella typhimurium* bacterial cells is discussed in [27]. Another smartphone fluorescence microscope design based on a low-cost inverted laser was showcased in [28]. Using this design, the authors successfully detected the *Trypanosoma cruzi* pathogen, which is responsible for Chagas disease. Furthermore, the authors in [29] have reported the utility of a smartphone-based fluorescence microscope for the rapid quantification of microplastics from environmental samples. This device can potentially serve as an initial assessment tool for the quantitative assessment of microplastics in remote areas with inadequate access to expensive resources. Another recent study [30] reports the use of a custom-designed smartphone fluorescence microscope to read the output of a CRISPR-Cas12a-based viral amplification assay for the development of an ultrasensitive and quantitative saliva test for COVID-19. Our group has also developed smartphone-based fluorescence microscopes that can work with multiple fluorophores, offer different magnification levels, and can be used to image and quantify leukocytes present in a sample [31,32,33,34].

Although SFMs have found various applications in the field of biomedical and modern healthcare, they suffer from various challenges when it comes to acquiring high-quality images due to a lack of sophisticated optics and digital resources. Significant improvements have been made to improve the quality of the smartphone-based microscope. Primarily, there are two methods to improve the quality of the image, i.e., using complex hardware or employing signal denoising techniques in the digital domain. Improving the power of the objective lens (numerical aperture), using high-intensity light sources, and illuminating the sample at an oblique excitation can improve the quality of the image, as discussed in the research [35,36]. Moreover, utilizing the excitation and emission filters with narrow bandwidths can potentially reduce the noise introduced to the image from the excitation source. In the digital domain, various methods have been developed, ranging from applying simple blurring techniques to complex deep learning-based methods to extract features and reconstruct images with low noise and high contrast values [37,38,39,40]. Employing deep learning-based methods requires extensive training of the models on huge datasets. In a typical deep learning-based framework, a different model is trained to extract the features from the image (extracting foreground and removing background noise), followed by an object detection and classification model trained separately. Introducing these steps and models with millions of weights further introduces computational complexity and increases the cost of the overall system, as more sophisticated smartphones are required. On the contrary, the smartphone-based microscope presented in this study relies on minimal optical components and low computational cost due to the simple denoising method and detection technique developed in the previous study [41]. Table S1 summarizes the comparison between the developed method and the existing techniques.

**Figure 1:**
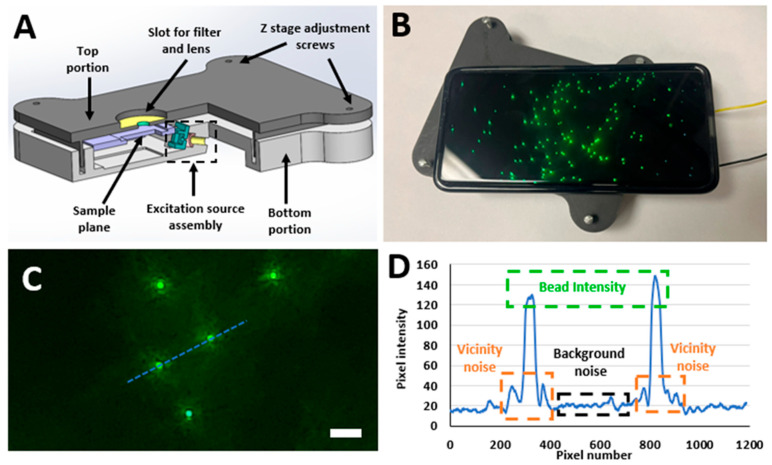
(**A**) The computer-aided design (CAD) model for the custom-built smartphone fluorescence microscope (SFM). (**B**) Samsung Galaxy S21 working with the 3D-printed prototype of the presented SFM for imaging fluorescent microbeads. (**C**) Green-fluorescent beads of 2 µm captured using the presented SFM at 4.5 V (scale bar = 8 µm). (**D**) The pixel intensity plot of the highlighted pixels indicated by the blue dashed line in (**C**) showcases the parameters of interest for the calculation of the image quality.

Moreover, efforts have been made to image the fluorescent particles at the nanoscale. Through the applications of powerful laser modules and complex optics, the researchers have been able to image particles as small as 50 nm [42,43,44]. Although much work has been carried out by researchers in the field of smartphone fluorescence microscopy, distinct SFM designs capable of imaging specimens in the sub-micron and nanometer range still require major novel breakthroughs in hardware and computational methods [45]. To address this limitation, we previously explored the design space of SFMs and compared the performance of SFMs equipped with different excitation modalities to identify the SFM design that produced the best quality images for fluorescent particles ranging from micrometers to sub-micron ranges in size [45]. The SFM design equipped with an oblique excitation modality, as shown in Figure 1, was found to produce the best quality of fluorescent images. We also discovered that excitation source intensity played an important role in the quality of captured images and identified the distinct optimal excitation voltage ranges required to produce the best images for fluorescent specimens of different sizes. Table S2 provides the optimal excitation voltages corresponding to the bead size. Bead intensity, bead vicinity noise, and background noise were picked as the parameters of interest to quantify the quality of the fluorescent images captured using this SFM design, as shown in Figure 1C,D. Based on these parameters of interest, the signal difference to noise ratio (SDNR) and contrast to noise ratio (CNR) for each captured image were calculated [46,47,48,49]. We also developed an automated algorithm that first measured the bead intensity, bead vicinity noise, and background noise in a captured fluorescent image and then calculated the SDNR and CNR values for that image [41].

In this paper, we further build on our past work and present the applications of the linear filtering algorithms for enhancing the imaging performance of SFMs. To achieve these goals, we employed a 3D signal Averaging technique based on linear filters of multiple kernel sizes (3 × 3 × 3, 7 × 7 × 7, 11 × 11 × 11, 15 × 15 × 15, and 21 × 21 × 21). Two different filtering approaches were picked: a simple 3D Averaging filter and a 3D Gaussian filter with three distinct σ values (1, 3, 5). These filters were then applied to the images of green, fluorescent beads (8.3, 2, 1, and 0.8 µm), and their effect was studied to identify the optimal filter parameters. Finally, the filters with the identified optimal parameters were applied to the images of fluorescently tagged leukocytes isolated from human peripheral blood, which were captured using the SFM shown in Figure 1. This validated the working of the SFM and these filters with actual biological samples in addition to the synthetic beads. The simplistic nature and easy application of these linear filters are their biggest strengths, as they allow quick and easy replication by other researchers who are looking to enhance the imaging performance of their setups. The details of the experimental setups and the conclusions drawn from this study are discussed in individual sections below.

## 2. Materials and Methods

### 2.1. Design of the Smartphone Fluorescence Microscope

The designed SFM is mainly composed of two portions, a top portion and a bottom portion, as shown in Figure 1A. The top portion of the SFM is mainly designed to contain the long pass filter for creating the darkfield background and the lens used for creating the necessary magnification. The long pass filter was acquired from Semrock, NY, USA (Product no: FF01-500/LP-23.3-D) and had a cut-off wavelength of 500 nm. The lens was acquired from Edmund Optics, NJ, USA (Stock #87-165) and had a focal length of 3.1 mm. This external lens and the internal lens of the smartphone’s camera module worked together to create the required optical magnification by forming a relay lens system. Additionally, the SFM’s top portion also contained four screw slots which were used for adjusting the SFM’s depth of focus to make sure it was focused on the desired imaging plane. The SFM’s bottom portion contained the custom-made excitation source (laser module), a slot for the battery and circuits, and the assembly used to house the fluorescent sample being imaged. The customized excitation source (laser module) was made by using a blue laser diode (Mouser electronics, TX, USA, Mfr. #: PLT5 450B), a lens with a focal length of 10 mm (Edmund optics, Stock #45-208) to focus light, and a 470 nm bandpass filter with a bandwidth of ∼40 nm (Chroma Inc, VT, USA, Product no. ET470/40x) to make sure that only the desired wavelengths of light reach the specimen placed on the sample plane. The laser beam formed from the laser module excited the sample plane at an oblique angle of 15°. This excited sample plane was then imaged using a smartphone of choice (Samsung Galaxy S21 Ultra, NJ, USA), which is placed on the top portion of the SFM. Figure 1B showcases a working prototype of the designed SFM. A quantitative feature comparison of this SFM with some previously published SFM designs by other researchers is shown in Table S3, and the details of calculating optical resolution are presented in the supplementary section I (A). Moreover, cost analysis has been added in Table S4, indicating that SFM costs in a fraction of benchtop fluorescence (Product # 89404-464 from VWR International, PA, USA) microscopes, which cost way over USD 15k.

### 2.2. Sample Preparation of Fluorescent Beads

Fluorescent beads (green) of different sizes (8.3, 2, 1, 0.8 µm) were used to test the performance of the designed SFM. The 8.3 µm, 2 µm, and 1 µm beads were purchased from Bangs Laboratories, IN, USA (Product # UMDG003, FSDG005, and FSDG004, respectively), whereas the smaller 0.8 µm beads (Product # HFP-0852-5) were obtained from Spherotech, IL, USA. 1X PBS (ThermoFisher, MA, USA, Catalog # 20012050) was used to dilute the acquired beads, and afterward, the designed SFM was used to image a 1 µL diluted sample for each of the four beads. Furthermore, all the experiments were repeated thrice to validate the obtained results and for quantification of the SFM’s imaging variance.

### 2.3. Leukocyte Extraction and Fluorescence Imaging

The Robert Wood Johnson Medical Hospital, New Brunswick, NJ, was used for the acquisition of human peripheral blood samples. This study was carried out with the approval of the Rutgers Institutional Review Board (IRB) committee (application # Pro2018002356). The hospital staff provided the leftover de-identified lactate test blood samples. Informed consent was waived by the IRB office owing to the collection of leftover blood samples, which were originally collected for patients’ routine care. Furthermore, all the experimental procedures followed the IRB protocol and ethical guidelines.

Totals of 100 µL sample of whole blood and 1 mL RBC lysis media (ThermoFisher, Cat # 00-4333-57) were poured into a centrifuge tube, and the resulting mixture was incubated for 10 min at 25 °C to lyse the red blood cells. Next, the lysis process was stopped by adding 2 mL 1X PBS (ThermoFisher, Catalog # 20012050) into the tube. This sample was then centrifuged for 5 min at 300× *g* to separate the leukocyte pellet and the supernatant. Pipetting was then performed to carefully remove this supernatant from the extracted leukocyte pellet. This leukocyte pellet was then lysed once again using the process described above to further purify it and remove any leftover red blood cells. The final extracted leukocytes were then suspended in 1x PBS.

SYTO 16 (ThermoFisher, Catalogue# S7578) green nuclear stain was used for fluorescent labeling of the extracted leukocytes. A total of 3 µL of SYTO 16 stain was diluted in 1 mL 1X PBS to prepare the fluorescent dye solution for staining the leukocytes. This fluorescent dye solution and the extracted leukocytes were poured into a 1.5 mL Eppendorf tube in an equal ratio and were then incubated away from light for 15 min at 25 °C. The fluorescently tagged leukocytes were then imaged using the SFM at multiple excitation voltages (4.3 V to 4.5 V) with a step size of 0.1 V. Similarly to the fluorescent beads, the imaging experiments for the leukocytes were also performed in triplicate.

### 2.4. AQAFI: Automated Quality Assessment of Fluorescent Images

Figure 1C,D showcase the three parameters of interest (Bead intensity, Bead vicinity noise, and Background noise) that were picked to quantitatively assess the quality of captured fluorescent images. Bead intensity corresponds to the intensity of the pixels in the image space that are illuminated by the fluorescence of a single bead/leukocyte. The vicinity noise relates to the region around the bead that was illuminated due to the dispersion of the light from the fluorescent particle (bead/leukocyte). Background noise constitutes the mean intensity of pixels present in the image background. These pixels are associated with the unwanted noise in the SFM excitation source that could not be filtered away by the bandpass filter. These parameters provide a coherent and uniform signal strength quantification at each bead level and were selected over PSNR or similar metrics as they require reference images, which is not practical for real-time SF imaging. Moreover, it was observed that each bead exhibits a slightly different shape of vicinity noise due to the scattering of the optical signal. Once these three parameters are quantified, two image quality assessment metrics, signal difference to noise ratio (SDNR) and contrast to noise ratio (CNR), are computed based on Equations (1)–(3) [41,46,47,48,49].

(1) $$SDNR=\frac{Bead\;Intensity-Vicinity\;Noise}{Background\;Noise}$$

(2) $$Contrast=\frac{Bead\;Intensity-Vicinity\;Noise\;}{Vicinity\;Noise}$$

(3) $$CNR=\frac{Contrast\;}{Background\;Noise\;}$$

To avoid the manual and tedious calculation process of these parameters using ImageJ v1.54h as employed in [45] and explained in the supplementary section II (B), we developed an automated quality assessment algorithm named AQAFI [41]. As shown in the supplementary section II, obtaining the count of beads in an image requires a series of manual steps, resulting in a tedious process. However, AQAFI automatically applies a multistep process to first measure the three parameters of interest (bead intensity, vicinity noise, and background noise) and then subsequently computes the values of SDNR and CNR for that image. The steps involved in this process are briefly described below:

1. In the first step, AQAFI uses a state-of-the-art feature detection algorithm (Fast Feature Detection) to identify the features or pixels associated with each bioparticle/bead. The fast feature detection algorithm classifies a pixel as a feature or corner if the intensity of that pixel is either higher or lower than a two-thirds majority of pixels in the Bresenham circle.
2. As the beads/leukocytes of multiple sizes were evaluated in this study, multiple features could be associated with a single bead/leukocyte. Once these features are identified in Step 1, an agglomerative clustering technique is used to find the center point of these small clusters, hence providing the location of the bioparticle/bead in the image space.
3. Once the location of each bead/leukocyte is known, bead intensity is computed by taking the Average of the pixel intensity values that are above the 99th percentile in a square area surrounding the particle.
4. The vicinity noise is estimated by first removing the bead intensity pixels in the selected square area of step 3 and then taking an average of the remaining pixel intensities.
5. The background noise is estimated by first removing all the pixels in the selected square area and then taking the mean of the other pixels.
6. Once these parameters are estimated, the SDNR and CNR value for each bead/leukocyte is calculated using Equations (1)–(3). AQAFI also reports the overall SDNR and CNR value of the image by computing the mean of the SDNR and CNR values of all the particles in an image. The detailed working algorithm of AQAFI can be found in [41].

### 2.5. Image Quality Enhancement Using Signal Averaging

Fluorescent images captured using the SFM at multiple excitation voltages (4.3 V to 4.5 V) were picked for algorithmic quality enhancement by noise reduction. In the spatial domain, the pixels associated with the background and vicinity noise can be considered as the high-frequency components scattered over the entire image. To conserve information gain, improve visibility, and remove these high-frequency components, we employed linear filtering methods and evaluated their effectiveness. The filtering techniques evaluated in this study are the 3D Average filter and the 3D Gaussian filters of multiple kernel sizes. The details of the process and an example of a 3 × 3 × 3 filter are shown in the supplementary section I. The application of these filters treats the color channels as three dimensions and averages the color values, resulting in a grayed image. To show the effect of filtering, the resulting images are false colored green. Figure S1 shows the output of the overall process of the 3D filtering operation and false coloring. Figure S2 showcases the detailed operation of image filtering using a 3D Averaging filter of size 3 × 3 × 3 applied to a single bead of 8.3 µm. The image also shows how the kernel is applied at a single channel (green) and how its output is calculated. Moreover, for reference, the calculations are also presented when the filter is applied to a particular pixel location.

#### 2.5.1. Three-Dimensional Averaging Filter

**Figure 2:**
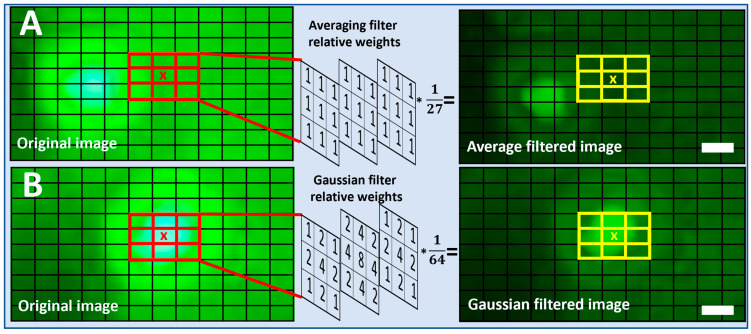
(**A**) Representative process showcasing the application of a simple Averaging filter for the quality enhancement of an SFM image (captured at 4.5 V) containing 8.3 µm beads (scale bar = 6 µm). (**B**) Representative process showcasing the application of a Gaussian filter for the quality enhancement of an SFM image (captured at 4.5 V) containing 8.3 µm beads (scale bar = 6 µm). ‘*’ Represents the multiplication operation.

Moving average is the simplest noise reduction filter used to remove the high-frequency components in an electrical signal. In the spatial domain, image filtering is achieved by the convolution operation. To apply the Averaging filter at a particular pixel location, the values in a kernel of size (m × m × m) carrying equal weights are first multiplied by the selected pixel and its surrounding pixels. The resultant is then added and averaged to obtain the single value as shown in Figure 2A. Equation (4) below summarizes the average filtering operation:

(4) $$g\left(x,y,z\right)=\sum_{u}\sum_{s}\sum_{t}k\left(s,t,u\right)f(x-s,y-t,\;z-u),$$

where $g\left(x,y,z\right)$ the filtered pixel, $f\left(x-s,y-t,\;z-u\right)$ is the original, and k(s,t,u) is the filer weights. In this study, we evaluate the performance of a 3D Averaging filter with five kernel sizes (3 × 3 × 3, 7 × 7 × 7, 11 × 11 × 11, 15 × 15 × 15, and 21 × 21 × 21). Unless otherwise specified, all the Averaging filters presented in this study are assumed to be 3D filters.

#### 2.5.2. Three-Dimensional Gaussian Filter

A Gaussian filter is another technique from the linear filtering methods. The 3D Gaussian filter also removes the high-frequency components by acting as a low-pass filter. Similarly to an Averaging filter, Gaussian filtering is achieved by applying a convolution operation. The weights of the 3D Gaussian kernel come from a 3D Gaussian distribution instead of the same weights in all the kernel cells. To apply a Gaussian filter at a particular pixel location, the values in a Gaussian kernel $K_{\sigma }$, as in Equation (6) of size (m × m), are first multiplied by the selected pixel and its surrounding pixels. The resultant is then added and averaged to achieve the single value as shown in Figure 2B. Equations (5) and (6) below summarize the Gaussian filtering operation:

(5) $$K_{\sigma }\left(x,y,z\right)=\frac{1}{{\left(2\pi \sigma^{2}\right)}^{3/2}}e^{-(\frac{x^{2}+y^{2}+z^{2}}{2\sigma^{2}})},$$

(6) $$g\left(x,y,z\right)=\sum_{u}\sum_{s}\sum_{t}k\left(s,t,u\right)f(x-s,y-t,\;z-u),$$

where $g\left(x,y,z\right)$ is the filtered pixel, $f\left(x-s,y-t,z-u\right)$ is the original, and K(s,t,u) are the 3D Gaussian filter weights. A Gaussian filter can be characterized by the size of the kernel and the standard deviation. As the filter performs the smoothing operation, the greater the standard deviation, the greater the smoothing effect. In this study, we evaluate the performance of the 3D Gaussian filter with five kernel sizes (3 × 3 × 3, 7 × 7 × 7, 11 × 11 × 11, 15 × 15 × 15, and 21 × 21 × 21) three levels of standard deviation σ (1, 3, 5). We applied each kernel with these varying values σ and studied its effects on image quality enhancement. Unless otherwise specified, all the Gaussian filters presented in this study are assumed to be 3D filters.

### 2.6. Assessing Spatial Resolution and Data Retention Rate

The application of the Gaussian and Averaging filters may result in blurring effects at the boundaries of the beads or leukocytes. As a result, in a concentrated sample, the beads may merge into a single shape, hence reducing the efficacy of the overall filtering operations. To evaluate this effect, we estimated the size of beads by drawing the circle enclosing the beads. We also estimated the increase in the spatial resolution between different beads by calculating the distance between the boundaries of the enclosed circles. Another important factor in analyzing the performance of the filtering methods is the data retention rate. Which measures the number of beads detected by the AQFI or ImageJ before and after the application of filtering techniques. The detailed process of counting beads using ImageJ is listed in the supplementary document in section II (B).

## 3. Results

### 3.1. Quality Enhancement of Fluorescent Bead Images

The SFM was able to successfully capture green fluorescent beads of four different sizes (8.3, 2, 1, and 0.8 μm) at excitation voltages ranging from 4.3 to 4.5 V. The quality of these captured images was enhanced by the application of Averaging and Gaussian filters of various kernel sizes (3 × 3 × 3, 7 × 7 × 7, 11 × 11 × 11, 15 × 15 × 15, and 21 × 21 × 21). The results obtained for each of these filters are discussed in the sections below:

#### 3.1.1. Three-Dimensional Averaging Filter

**Figure 3:**
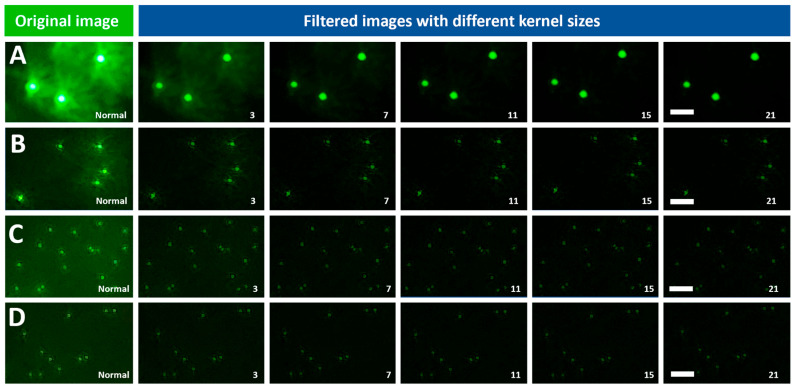
Visual representations of the image quality enhancement of different bead sizes imaged using the SFM at 4.5 V after the application of Averaging filters of various kernel sizes (scale bar = 20 µm). Bead sizes of (**A**) 8.3 µm, (**B**) 2 µm, (**C**) 1 µm, (**D**) 0.8 µm. Note: The numbers on each picture represent 3 × 3 × 3 to 21 × 21 × 21 filters.

**Figure 4:**
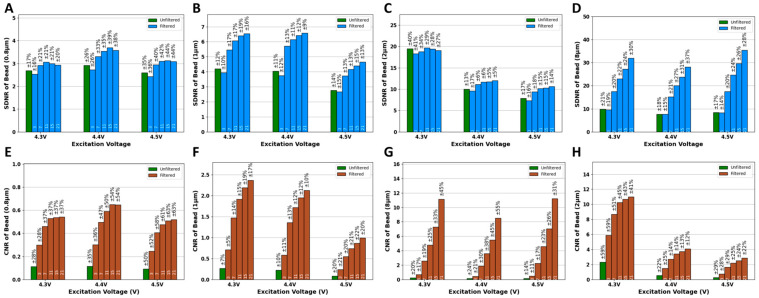
SDNR and CNR plots of different bead sizes before and after applications of Averaging filters at different kernel sizes. (**A**–**D**) SDNR plots for 8.3, 2, 1, 0.8 µm, respectively. (**E**–**H**) CNR plots for 8.3, 2, 1, 0.8 µm, respectively. Note: The numbers inside each bar picture represent 3 × 3 × 3 to 21 × 21 × 21 filters.

The application of Averaging filters of various kernel sizes improved the quality of the images of the four fluorescent beads (8.3, 2, 1, and 0.8 μm). Figure 3A–D shows the visual quality enhancement of the 8.3, 2, 1, 0.8 μm beads imaged at 4.5 V after the application of an Averaging filter of multiple kernel sizes, respectively. In addition to visual inspection of the improvement in image quality, it is also important to quantitatively assess this improvement. To do so, the automated algorithm (AQAFI) discussed in previous sections was used, which quantified the SDNR and CNR of a particular image. Figure 4A–D show the corresponding improvements in the SDNR, and Figure 4E–H show the improvements in the CNR for the four fluorescent beads (8.3, 2, 1, and 0.8 μm) before and after applications of Averaging filters, respectively.

#### 3.1.2. Three-Dimensional Gaussian Filter

**Figure 5:**
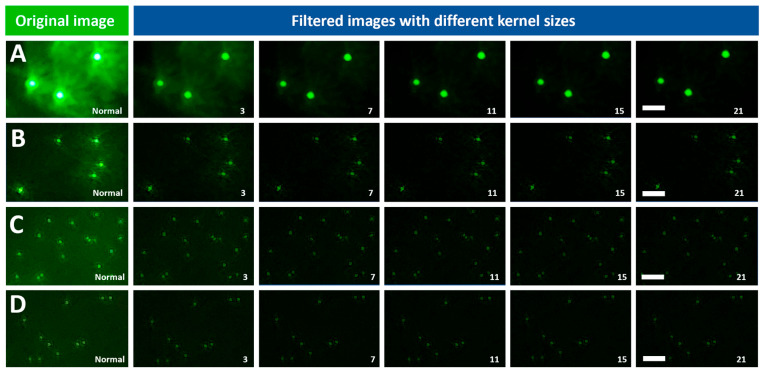
Visual representations of the image quality enhancement of different bead sizes imaged using the SFM at 4.5 V after the application of Gaussian filters (sigma = 5) of various kernel sizes (scale bar = 20 µm). Bead sizes of (**A**) 8.3 µm, (**B**) 2 µm, (**C**) 1 µm, (**D**) 0.8 µm. Note: The numbers on each picture represent 3 × 3 × 3 to 21 × 21 × 21 filters.

**Figure 6:**
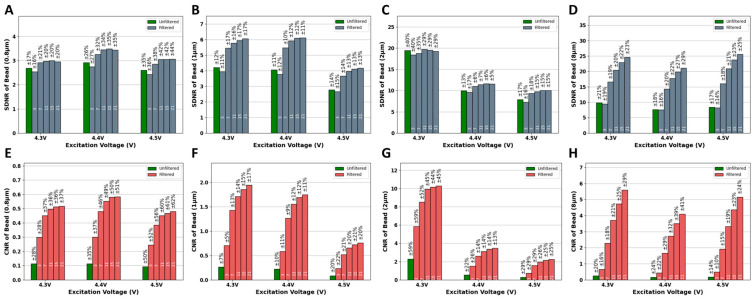
SDNR and CNR plots of different beads sizes before and after applications of a Gaussian filter (σ = 5) of various kernel sizes. (**A**–**D**) SDNR plots for 8.3, 2, 1, 0.8 µm, respectively. (**E**–**H**) CNR plots for 8.3, 2, 1, 0.8 µm, respectively. Note: The numbers inside each bar picture represent 3 × 3 × 3 to 21 × 21 × 21 filters.

The application of a Gaussian filter (σ = 5) of various kernel sizes improved the quality of the images of the four fluorescent beads (8.3, 2, 1, and 0.8 μm). Figure 5A–D of the 8.3, 2, 1, 0.8 μm beads imaged at 4.5 V after the application of Gaussian filters (σ = 5) of various kernel sizes, respectively. Similarly to the Average filter, AQAFI was used to quantify the improvement in the SDNR and CNR of an image before and after the application of the Gaussian filters. Figure 6A–D show the corresponding improvements in the SDNR and Figure 6E,F show the improvements in the CNR for the four fluorescent beads (8.3, 2, 1, and 0.8 μm) before and after applications of Gaussian filters (σ = 5) of various kernel sizes, respectively. Moreover, the quantitative improvement for the SDNR and CNR values after the application of Gaussian filters with σ (1, 3) is shown in Figures S3 and S4.

## 4. Quality Enhancement of Leukocyte Images

**Figure 7:**
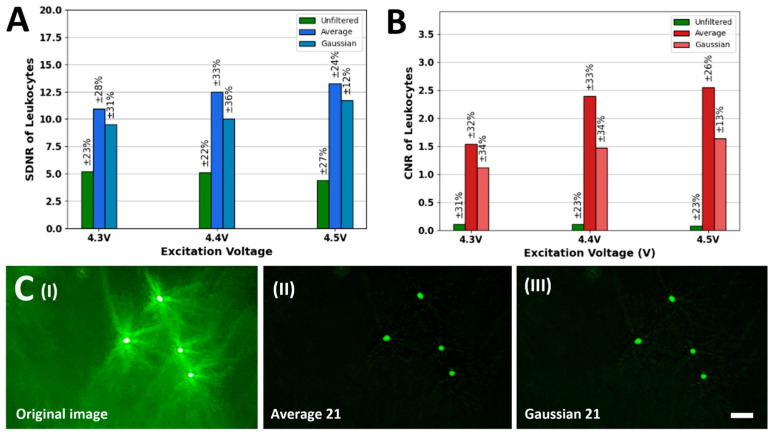
(**A**) SDNR plots for the fluorescently tagged leukocyte images enhanced using an Averaging filter (kernel size = 21 × 21 × 21) and a Gaussian filter (σ = 5, kernel size = 21 × 21 × 21). (**B**) Corresponding CNR plots. (**C**) (**I**) Original unprocessed fluorescently tagged leukocyte image captured using the SFM at 4.5 V. (**II**) Enhanced quality leukocyte image produced by the application of an Averaging filter (kernel size = 21 × 21 × 21) on (**I**). (**III**) Enhanced quality leukocyte image produced by the application of a Gaussian filter (σ = 5, kernel size = 21 × 21 × 21) on (**I**) (scale bar = 25 µm). Note: The numbers on each picture represent 3 × 3 × 3 to 21 × 21 × 21 filters.

Based on the data shown in Figure 3, Figure 4, Figure 5 and Figure 6 we concluded that for both average and gaussian filters, a kernel size equal to 21 × 21 × 21 was best for enhancing the quality of fluorescent images of the four beads (8.3, 2, 1, and 0.8 μm) at all excitation voltages. Thus, average and Gaussian (σ = 5) filters with a kernel size equal to 21 × 21 × 21 were applied to the leukocyte images captured using the SFM. Figure 7A showcases the improvement in SDNR after the application of the filters, whereas the corresponding increase in the CNR values is shown in Figure 7B. The visual improvement in the image quality of the leukocytes shown in Figure 7C(I) after the application of the Average filter is shown in Figure 7C(II), whereas Figure 7C(III) showcases the improved image after the application of a Gaussian filter (σ = 5).

## 5. Discussion

The Averaging and Gaussian filters were applied to the images of the fluorescent beads (8.3, 2, 1, and 0.8 μm) captured at excitation voltages ranging from 4.3 to 4.5 V. Figure 3 showcases both the original images of the fluorescent beads (8.3, 2, 1, and 0.8 μm) captured at 4.5 V, and the reconstructed images after the application of Averaging filters of various kernel sizes. From a visual perspective, we can observe that the application of Averaging filters of different kernel sizes results in a different final image. The bigger the kernel size, the better the performance regarding the reduction in the background and vicinity noise. The kernel size 21 × 21 × 21 performed the best, as can be seen from the final reconstructed images for the 8.3 and 2 μm beads. Almost all the noise (background and vicinity) was removed from the image, and the original beads could be seen more clearly discerned from the background. In addition to the bigger beads, the application of the averaging filters also improved the quality of the images captured for the smaller 1 and 0.8 μm beads. This improvement does not look that significant because, in contrast to the 8.3 and 2 μm beads, the smaller beads do not have much noise (background and vicinity) in the original unfiltered images to begin with. The application of the Gaussian filter (σ = 5) resulted in trends similar to that of an Averaging filter, as can be seen from Figure 5. The kernel size 21 × 21 × 21 gave the best performance and drastically reduced the observed noise (background and vicinity) in the captured images. Similarly to the Averaging filter, the effect of the application of Gaussian filters is more apparent in the bigger beads (8.3 and 2 μm) since they contain more noise compared to the smaller ones 1 and 0.8 μm. To further understand the effect of 3D filtering operations, we also performed a 2D filtering operation on the 8.3 µm single bead image, as shown in Figure S5 (we performed this additional analysis to compare both methods). It was observed that the application of 2D Averaging or Gaussian filters simply blurs the original image while the overall background and vicinity noise remain consistent. A detailed blurring effect of 2D kernels on fluorescent images can be observed in Figure S6. A set of 2D kernels was applied to beads of all sizes discussed earlier, and it was observed that filters having sizes bigger than the bead size significantly blur the image, reducing the overall information content in the image. The extraordinary performance of 3D filters can be attributed to the filtering operating across the channel dimension, as the red and blue channels contain minimal noise values, as shown in Figure S7A. When averaged over channel dimensions, the filters produce lower noise and bead intensity values, as red and blue channels contain lower intensity values. However, the effect is not the same when each channel is treated as a single matrix and a 2D Gaussian or Averaging filter is applied.

It can also be observed from Figure S2A that, since the red or blue channels contain minimal noise values, they can be considered as the noiseless image and false-colored to extract the bead metrics. However, this is not true, as the images are captured by a standard smartphone; only the beads of a larger size and with the highest fluorescence intensity are captured in all three RGB channels. On the other hand, the smaller size beads or beads with low fluorescence intensity are either partially captured or not captured at all in red or blue channels, as shown in Figure S7. Thus, using red and blue channels as the source of information may lead to information loss.

In addition to assessing the impact of the application of these filters on the visual quality of the SFM images, we also evaluated the effect of these filters in a more quantitative way on the parameters of interest (bead intensity, vicinity noise, and background noise). Figures S8–S10 showcase the effect of the application of these filters on the bead intensity, vicinity noise, and background noise, respectively. For all three parameters, the application of Averaging and Gaussian filters results in a decrease in their mean values as compared to their values in the original unfiltered images captured at any particular voltage. The effect can also be observed in the intensity profiles of 8.3 µm beads before and after applications of filters in Figure S11. Furthermore, the application of Averaging and Gaussian filters with a kernel size of 3 × 3 × 3 results in a huge drop in the values of the parameters of interest when compared to the original unfiltered image. This is why in Figure 4 we see a slight drop in the SDNR values for images corrected with filters of kernel size equal to 3. The reduction in the background noise alone is not enough to compensate for the reduction in the difference between bead intensity and vicinity noise. Thus, to see better quantitative SDNR results, filters with larger kernel sizes can be used, which results in a much larger reduction in background noise and an increase in the SDNR. For the CNR, we have both background noise and vicinity noise in the denominator; therefore, even the filters with smaller kernel sizes result in improving the CNR compared to unfiltered images, as seen in Figure 6.

The application of progressively larger kernel sizes (7 × 7 × 7, 11 × 11 × 11, 15 × 15 × 15, and 21 × 21 × 21) further reduces the mean values of the parameters of interest. This difference in the performance of bigger kernels is not as significant as the one observed when going from an unfiltered image to the one produced after the application of a filter with a kernel size equal to 3 × 3 × 3. Thus, although using kernel sizes bigger than 21 × 21 × 21 may slightly further improve the image quality by minutely reducing the noise (vicinity and background) in the image, it would also enhance the risk of losing out on meaningful data by further reducing the bead intensity, which is undesirable.

After testing the Average and Gaussian filters on the images of fluorescent beads (8.3, 2, 1, and 0.8 μm), these filters were also applied to the images of fluorescently tagged human peripheral blood leukocytes. For both Average and Gaussian filters, only a kernel size of 21 × 21 × 21 was applied since it performed the best based on the observed data. Since the leukocyte subpopulations can range from anywhere between 6 and 15 μm, a behavior similar to that of 8.3 μm beads was expected after the application of Average and Gaussian filters. Figure 7 showcases the effect of the application of the Average and Gaussian filters on the images of a fluorescently tagged leukocyte sample. As expected, the application of both the Average and Gaussian filters significantly reduced the observed noise in the image and improved the SDNR and CNR values. Furthermore, as shown in Figure S12, this improvement in the image quality was also accompanied by changes in bead intensity, background noise, and vicinity noise. Similarly to the trend observed in the beads imaging experiments, the Averaging filter again outperforms the Gaussian filter when it comes to purely quantitative improvement in the SDNR and CNR values. The reason behind that is that the background and uniform noises in the image are high-frequency. The Averaging filter assigns equal weights to them, resulting in the flattening of local variations. On the other hand, the Gaussian filter takes the weighted averages of the background and vicinity noise pixels, and the high-frequency components are not equally suppressed. This results in a higher average of the background and vicinity noise values and a reduction in the SDNR and CNRs. Figure S13 showcases the application of Average and Gaussian filters on two other samples of fluorescently tagged leukocytes, and a similar improvement is observed in image visual quality, SDNR, and CNR values, along with changes in bead intensity, background noise, and vicinity noise. Moreover, we summarized the contrast, noise, and CNR values in Tables S5–S7 to provide a clear understanding of the effectiveness of filtering methods on beads and leukocyte images.

To showcase the universal nature of the linear filters and how researchers using fluorescence microscopes of different brands and models can use them, we applied these linear filters to fluorescence images of 8.3 µm green beads imaged using a benchtop fluorescence microscope. As seen in Figure S14, the application of these linear filters enhanced the quality of the image by reducing the background and vicinity noise. We can see that the improvement in CNR is much better compared to SDNR, and this is because the original unfiltered image contained very low background noise but slightly higher vicinity noise. As explained above, a reduction in the vicinity noise manifests in the form of enhanced CNR, and thus, we see better improvement for the CNR compared to SDNR. Moreover, when applying Gaussian or Averaging filters, a key factor may be observed where the size of the beads may increase due to the smoothing effect. We observed an approximate increase in the size of ~40 pixels of 8.3 µm beads after the application of the filters, as can be observed in Figure S15. We also performed the effect of the filtering on perspective improvement in the spatial resolution of the microscope and observed an approximate increase of 6 µm in spatial resolution, as shown in Figure S16.

We also performed the image data loss analysis on the fluorescent images before and after applying the filters. We compared the information retention rate of the applied filters and quantified the number of beads retained using both ImageJ and AQAFI. We observed a minimum retention rate of over 95% for the beads of size 0.8 μm, as some of the beads were lost as they were out of focus and were present at the edges of the FOV. Therefore, the bead intensity signal was significantly weak, resulting in a loss. On the other hand, the data retention rate for beads of bigger sizes, i.e., >2 µm, is above 98%. Reconstructed filtered images from all kernel sizes retained the original meaningful data, and the slight variance in the number of observed beads can be attributed to the manual processing errors associated with bead counting using ImageJ. Thus, although the bead intensity is reduced after the application of the averaging and Gaussian filters, the corresponding reduction in the background noise and vicinity noise is much greater. Tables S8–S11 summarize the data retention rates for all bead sizes at different excitation voltages discussed in the previous sections.

The application of the developed average and Gaussian filters on fluorescent images (beads and leukocytes) captured using an SFM improves their quality by reducing the image noise. These filters also reduce the operational complexity of the SFMs by obviating the need to adjust the excitation source intensity based on the fluorescent specimen being imaged. The simplistic nature and ease of use are perhaps the biggest strengths of these filters, as they can be easily adapted to be used with a wide range of existing fluorescence microscope designs and enhance their imaging capabilities. In the future, we aim to broaden the scope of study by incorporating multicolor fluorophores, processing dynamic images, and training custom deep learning models for object detection, recognition, and tracking.

## Data Availability

The data related to this study will be available upon request, based on the funding agencies and the policies of Rutgers, The State University of New Jersey. A request can be made to the corresponding author.

## Supplementary Materials

The following supporting information can be downloaded at https://www.mdpi.com/article/10.3390/bios15070403/s1.
