# Supplementary material for: Enhancing Biomarker Detection and Imaging Performance of Smartphone Fluorescence Microscopy Devices
Source: Biosensors (Basel). 2025 Jun 21;15(7):403. doi: 10.3390/bios15070403 (PMC12293553; doi:10.3390/bios15070403)
Supplement: Supplementary file 1 [file biosensors-15-00403-s001.zip › biosensors-3635855-supplementary.pdf]

# Supplementary Information

## Enhancing Biomarker Detection and Imaging Performance of Smartphone Fluorescence Microscopy Devices

**Authors:** Muhammad A. Sami[1,2], Muhammad Nabeel Tahir[1,2], Umer Hassan*[1,3]

[1] Department of Electrical and Computer Engineering, School of Engineering, Rutgers, The State University of New Jersey, United States of America.

[2] These authors contributed equally to this work.

[3] Global Health Institute, Rutgers, The State University of New Jersey, New Brunswick, United States of America.

*Corresponding Author: umer.hassan@rutgers.edu (UH)

# I. Image Filtering Operations and Explanations

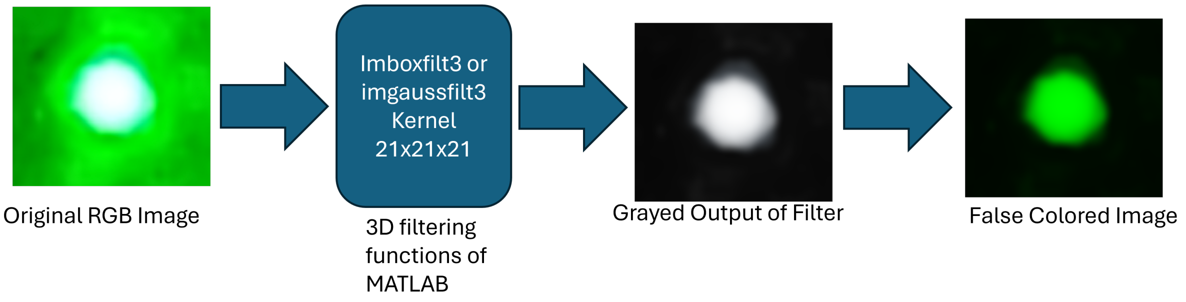


Fig S1: Image filtering operation explained with kernel of 21x21x21 applied to a single bead of 8.3µm.

Figure below shows the detailed operation of 3D filtering when applied to an RGB image. The image also shows the information in each channel by splitting the channel into R, G, and B respectively. Moreover, shows the process of how the filtering is performed. First the padding is applied to the original image by replicating the values on the border of the image in all three dimensions. Then the image filtering operation is performed to obtain the final output as shown in Fig. S2(C).

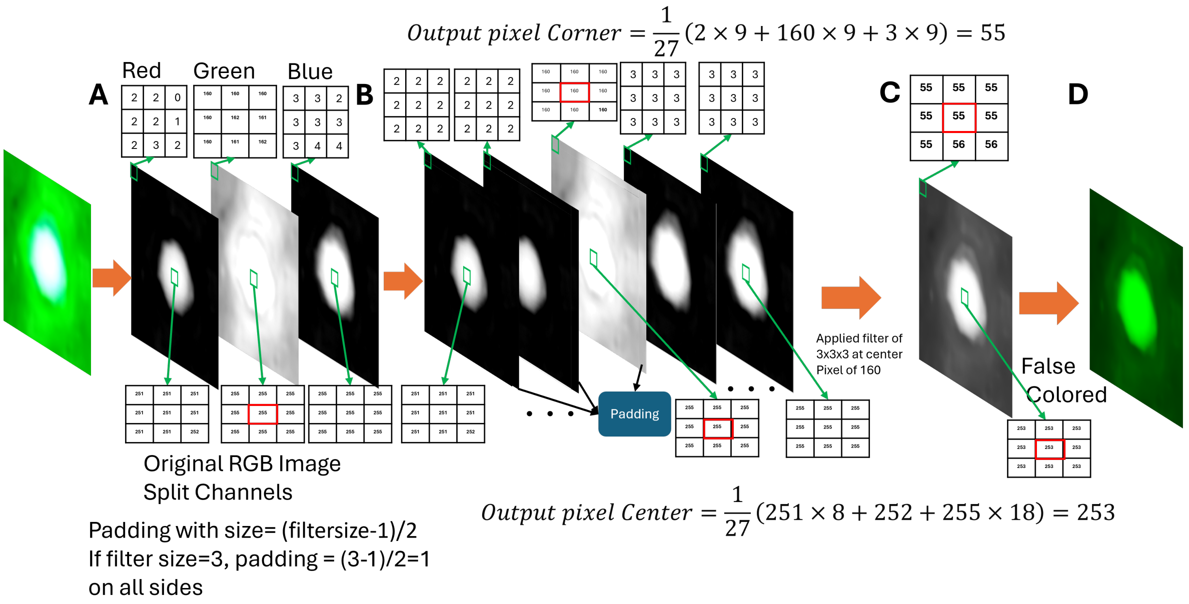


Fig. S2: Detailed computations of an averaged 3D filter operation with kernel 3x3x3 applied to the single bead image of size 8.3µm. (A) Original RGB image with split channels and matrices showing the values in a 3x3 kernel. (B) Padded RGB image with padding sizes mentioned in the figure and kernel values. (C) The output image of the 3D averaging filter with center pixel value highlighted in red. (D) False colored image.

# II. SUPPLEMENTARY METHODS

## A. *Imaging performance of SFM*

A Samsung Galaxy S21 Ultra was picked for capturing the images of fluorescent beads and leukocytes. A magnification factor of 2.16 was achieved for this SFM configuration based on the focal lengths of the external lens (3.1 mm) and the smartphone's internal lens (6.7 mm). The optical resolution of this SFM configuration came out to be 2.2 μm based on measurements obtained using a 1951 UASF [35]. Furthermore, the designed SFM was also able to successfully image the fluorescent beads and leukocytes as shown in Fig. 4, 6, and 7. The optimal excitation voltage ranges for the four beads are listed in Table S1.

## B. *Image data loss analysis*

The results shown in Fig. 3 and 5 indicate that the application of average and gaussian filters reduces the noise and thereby enhances the quality of captured images. In addition to the reduction in the noise intensity, the application of these filters also reduces the bead intensity. This is less critical for the higher-intensity beads, but the beads that are dim to begin with are at risk of getting lost because of the application of the average and Gaussian filters. Therefore, the quantity of the beads present in the original unfiltered images and the reconstructed images obtained after the application of the filters was measured to check for any detection loss. ImageJ was thus used to quantify the beads in the original images and the reconstructed filtered images. The steps to calculate the beads using ImageJ are listed below.

1. Open the image to be counted. If it is a color image (RGB), it will have to be converted to greyscale before proceeding. Check that you have set Edit → Options → Conversions to "scale when converting." Then, use Image → Type → 16-bit to convert to greyscale.
2. Once the image is in greyscale (8-bit or 16-bit), use Image → Adjust → Threshold (Cntl + Shift + T) to highlight all of the structures you want to count. To highlight, either use the sliders or use the "set" button to type in a known range of pixel intensities (if you want to threshold a whole set of images the same way, for instance).
3. If you have merged particles, Process → Binary → Watershed.
4. Once you have a binary image of the particles you wish to count, go to Analyze → Analyze Particles.

Tables S3-S6 showcase the number of beads present in the original unfiltered images and the ones present in the reconstructed filtered images coming after the application of averaging and Gaussian filters of different kernel sizes and σ values. Reconstructed filtered images from all kernel sizes retained the original meaningful data and the slight variance in the number of observed beads can be attributed to the manual processing errors associated with bead counting using ImageJ. Thus, although the bead intensity gets reduced after the application of the averaging and gaussian filters, the corresponding

reduction in the background noise and vicinity noise is much greater. It’s worth noting that a sample containing a smaller number of beads can be prepared by adjusting the concentration of the particles to avoid the beads getting too close and matching the spatial resolution of the microscope.

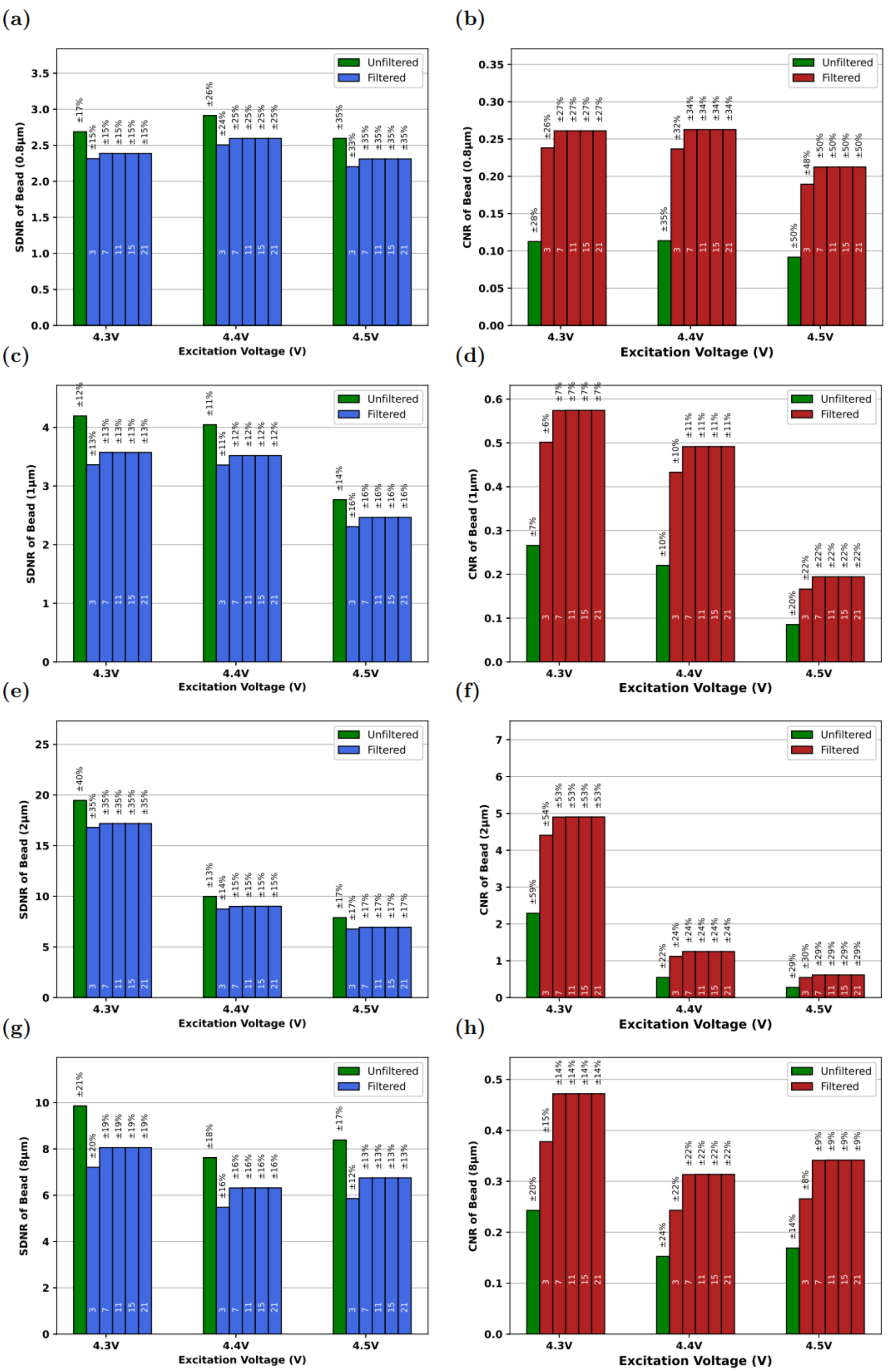


Fig S3. (A) SDNR plots for the 0.8 µm fluorescent bead images enhanced using a Gaussian filter (σ = 1) of various kernel sizes. (B) Corresponding CNR plots. (C) SDNR plots for the 1 µm fluorescent bead images enhanced using a Gaussian filter (σ = 1) of various kernel sizes. (D) Corresponding CNR plots. (E) SDNR plots for the 2 µm fluorescent bead images enhanced using a Gaussian filter (σ = 1) of various kernel sizes. (F) Corresponding CNR plots. (G) SDNR plots for the 8.3 µm fluorescent bead images enhanced using a Gaussian filter (σ = 1) of various kernel sizes. (H) Corresponding CNR plots. Note: The numbers inside each bar picture represent 3x3x3 to 21x21x21 filters.

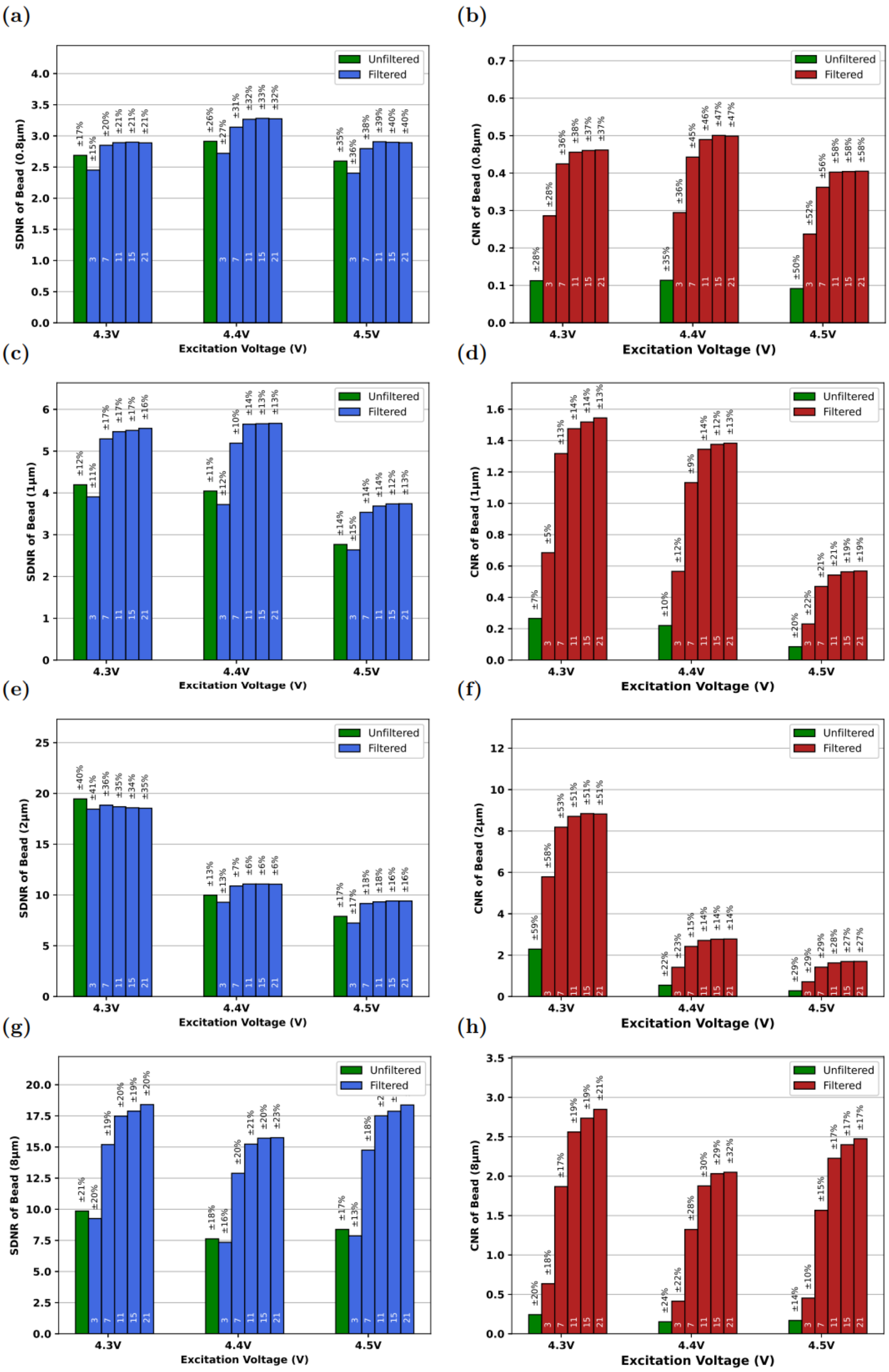


Fig S4. (A) SDNR plots for the 0.8 μm fluorescent bead images enhanced using a Gaussian filter (σ = 3) of various kernel sizes. (B) Corresponding CNR plots. (C) SDNR plots for the 1 μm fluorescent bead images enhanced using a Gaussian filter (σ = 3) of various kernel sizes. (D) Corresponding CNR plots. (E) SDNR plots for the 2 μm fluorescent bead images enhanced using a Gaussian filter (σ = 3) of various kernel sizes. (F) Corresponding CNR plots. (G) SDNR plots for the 8.3 μm fluorescent bead images enhanced using a Gaussian filter (σ = 3) of various kernel sizes. (H) Corresponding CNR plots. Note: The numbers inside each bar picture represent 3x3x3 to 21x21x21 filters.

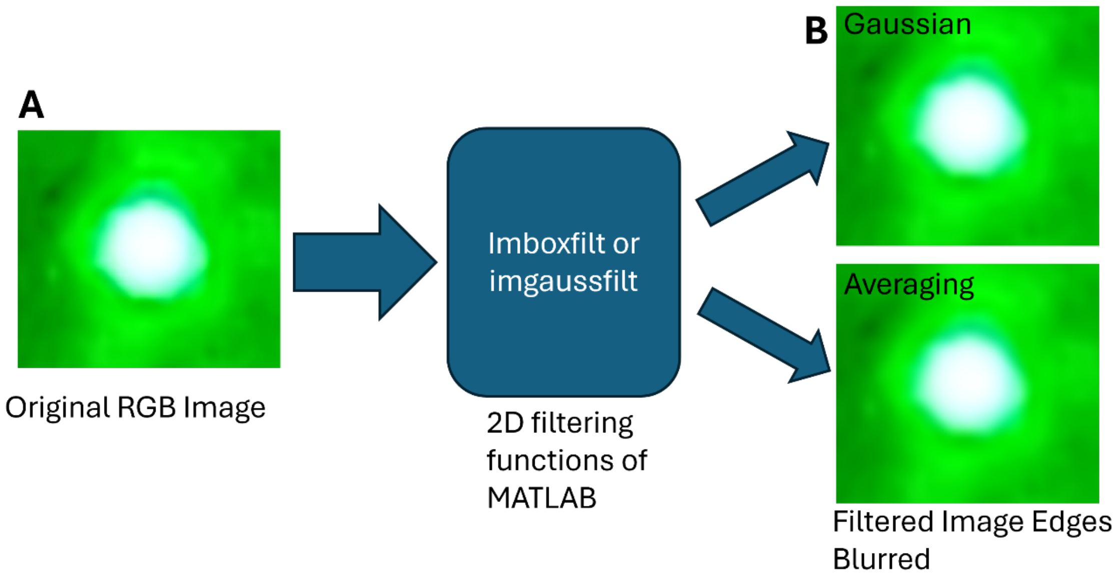


Fig S5. Effect of 2D filtering operation (Gaussian and Averaging) on a single bead of size 8.3µm with a kernel size of 21x21. (A) Original image of the bead. (B) Filtered image of the bead.

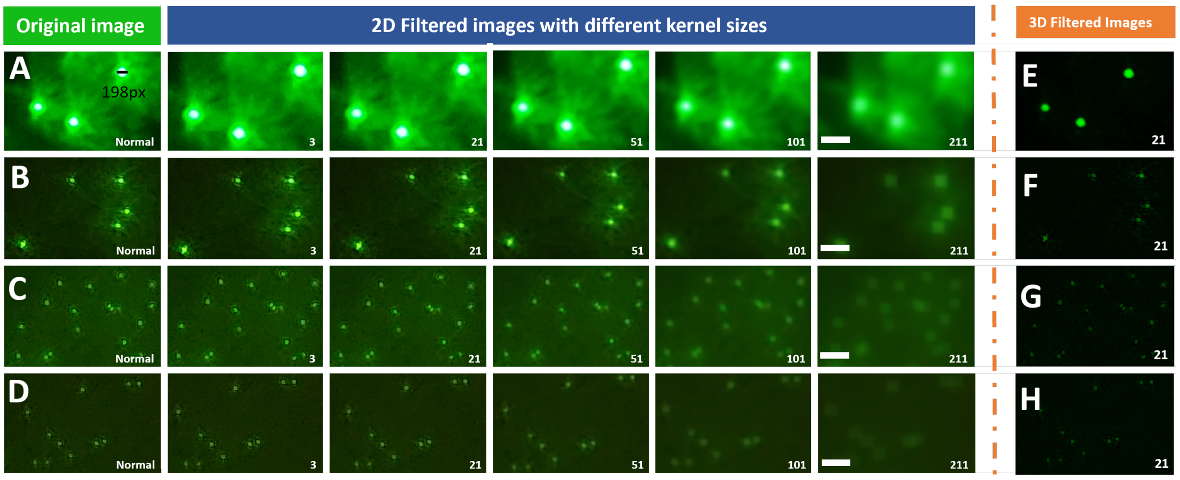


Fig S6. (A) The 8.3 µm beads imaged using the SFM at 4.5 V and results of the application of 2D Averaging filters of various kernel sizes (scale bar = 20 µm). (B) The 2 µm beads imaged using the SFM at 4.5 V and results of the application of 2D Averaging filters of various kernel sizes (scale bar = 20 µm). (C) The 1 µm beads imaged using the SFM at 4.5 V and results of the application of 2D Averaging filters of various kernel sizes (scale bar = 20 µm). (D) The 0.8 µm beads imaged using the SFM at 4.5 V and results of the application of 2D Averaging filters of various kernel sizes (scale bar = 20 µm). (E) Visual representation of the image quality enhancement of 8.3 µm beads imaged using the SFM at 4.5 V after the application of 3D Averaging filters with kernel 21x21x21. (F) Visual representation of the image quality enhancement of 2 µm beads imaged using the SFM at 4.5 V after the application of 3D Averaging filters with kernel 21x21x21. (G) Visual representation of the image quality enhancement of 1 µm beads imaged using the SFM at 4.5 V after the application of 3D Averaging filters with kernel 21x21x21. (H) Visual representation of the image quality enhancement of 0.8 µm beads imaged using the SFM at 4.5 V after the application of 3D Averaging filters with kernel 21x21x21. *Note: The numbers on each picture A-D represent 3x3 to 211x211 filters.*

The 2D averaging filter treats each color channels as a separate matrix and applies the filter on each channel separately. Resulting in a blurred image as shown in the Fig. S5 (B).

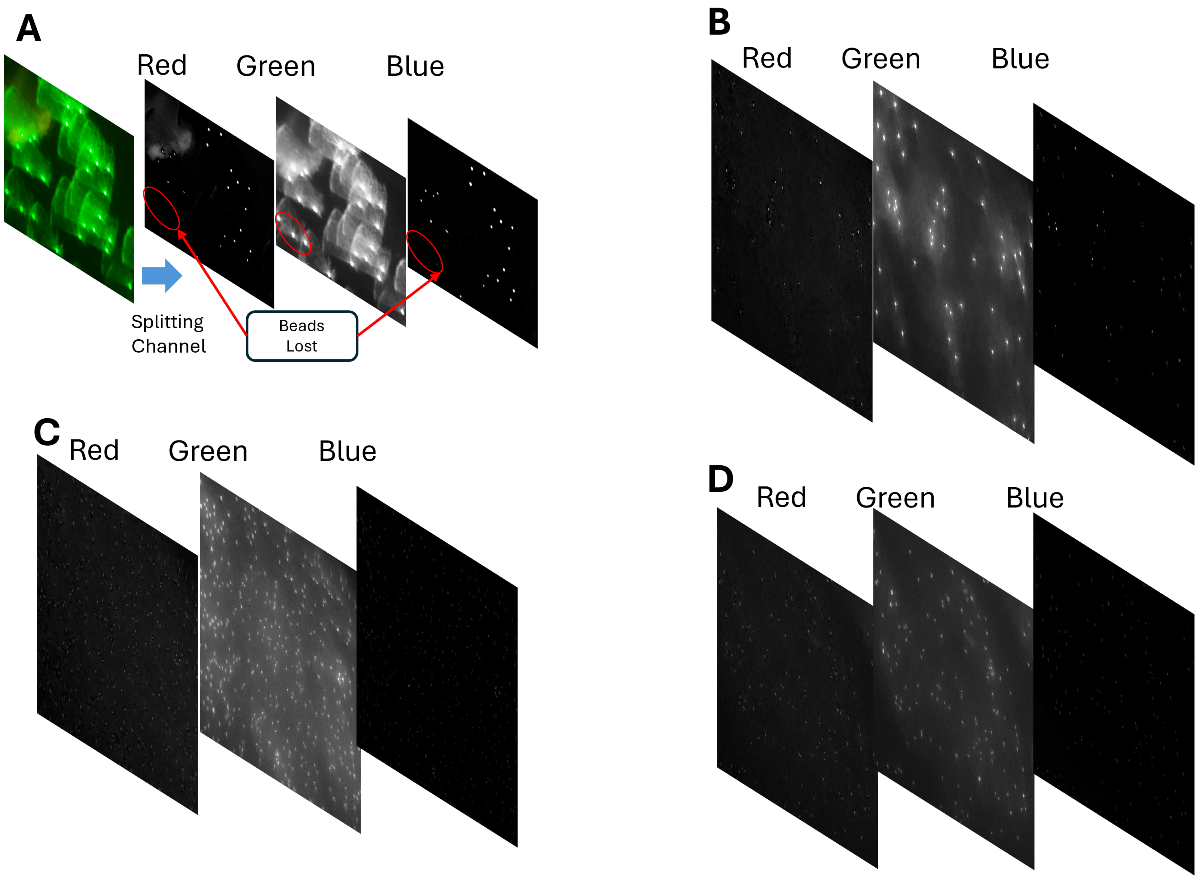


Fig S7. RGB channel information of different bead sizes. (A) Split RGB image of 8.3µm beads with missing information highlighted in red and blue channels. (B) Split RGB image of 2µm beads showing partial to missing information in red and blue channels. (C) Split RGB image of 1µm beads showing partial to missing information in red and blue channels. (D) Split RGB image of 0.8µm beads showing partial to missing information in red and blue channels.

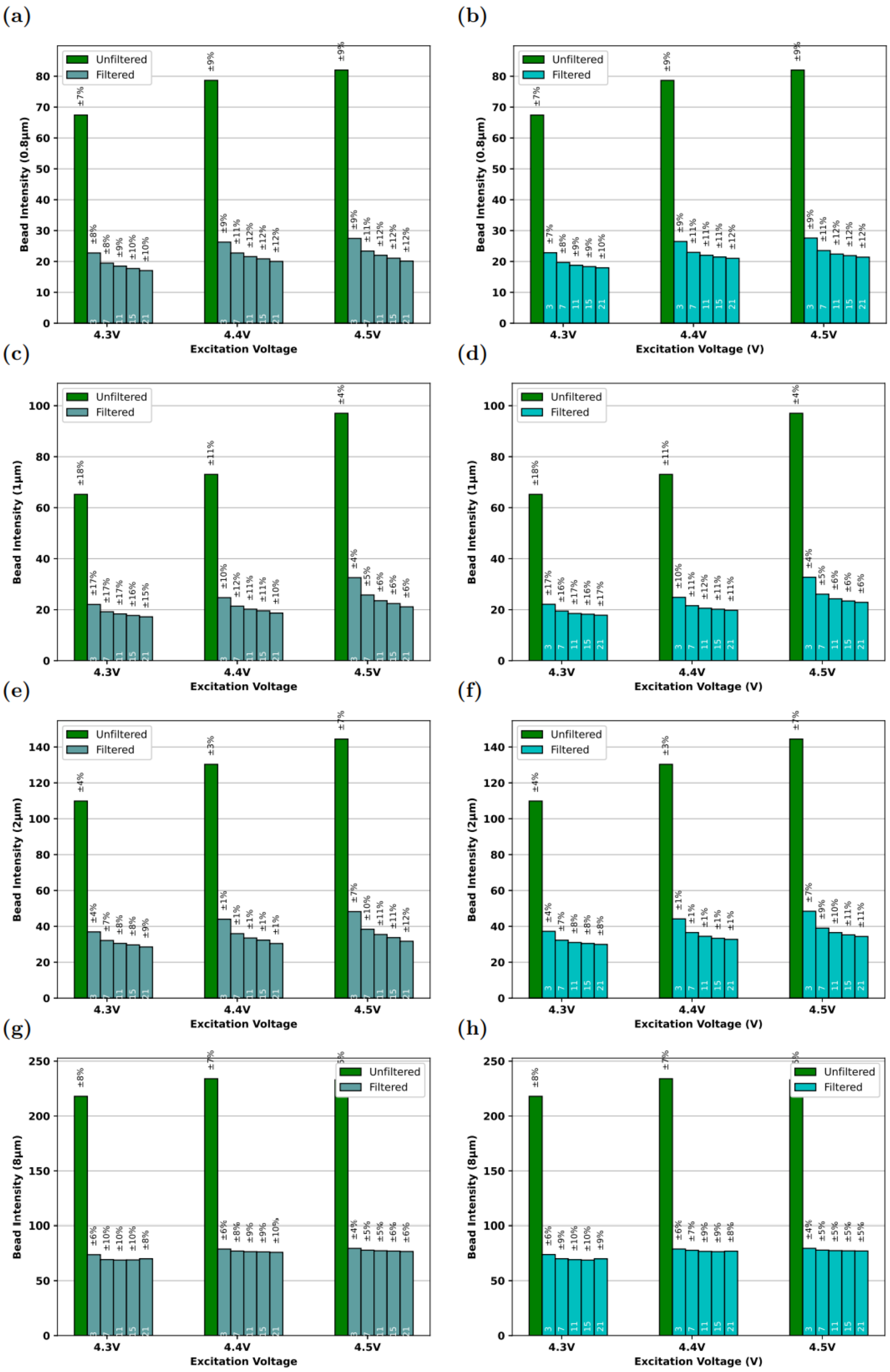


Fig S8. Bead Intensity plots after applying Averaging and Gaussian filters (σ = 5) with different kernel sizes: (a) Bead Intensity of 0.8 μm beads with Averaging filter. (b) Bead Intensity of 0.8 μm beads with Gaussian filter. (c) Bead Intensity of 1 μm beads with Averaging filter. (d) Bead Intensity of 1 μm beads with Gaussian filter. (e) Bead Intensity of 2 μm beads with Averaging filter. (f) Bead Intensity of 2 μm beads with Gaussian filter. (g) Bead Intensity of 8.3 μm beads with Averaging filter. (h) Bead Intensity of 8.3 μm beads with Gaussian filter. Note: Note: The numbers inside each bar picture represent 3x3x3 to 21x21x21 filters.

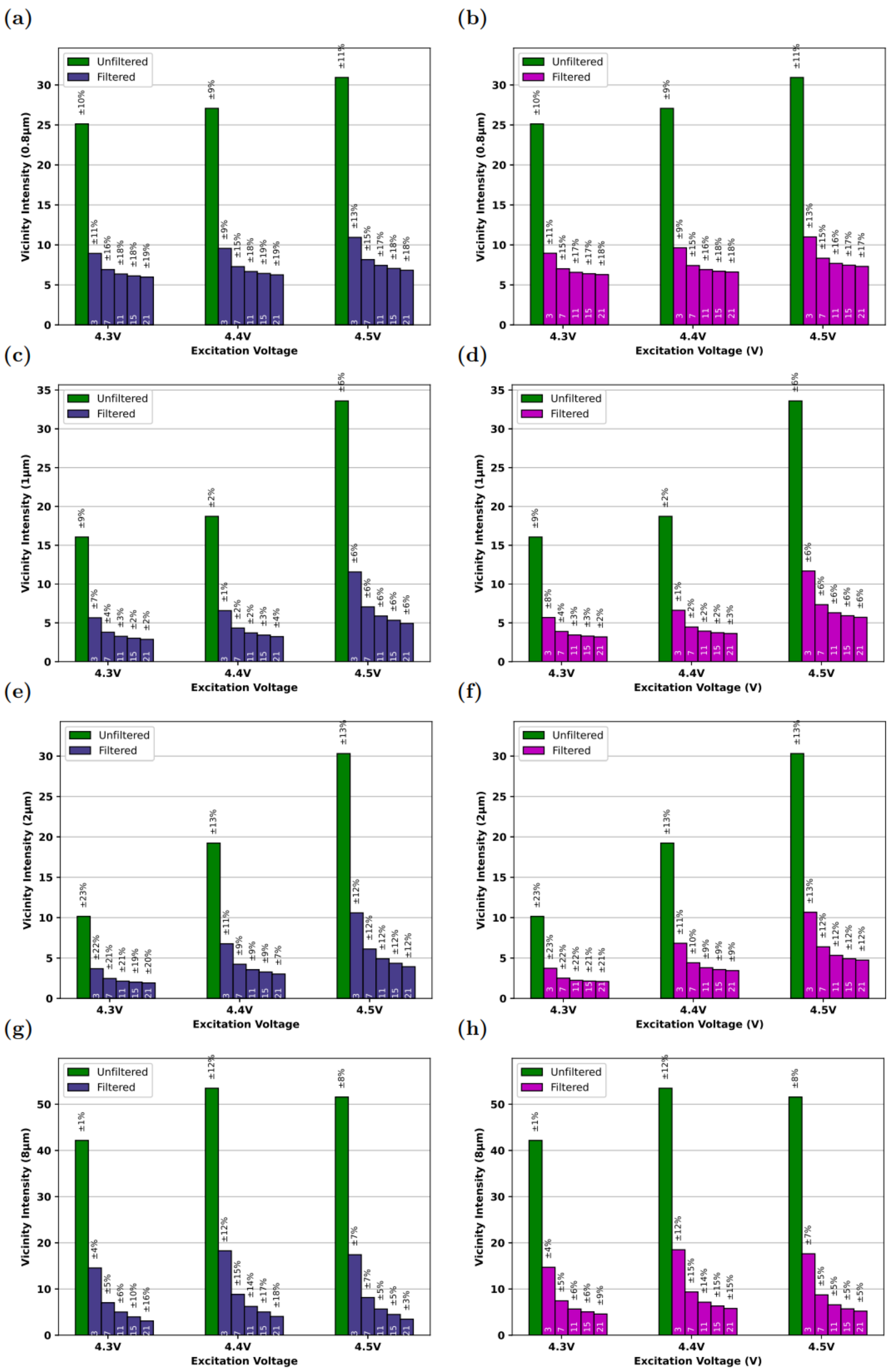


Fig. S9. Vicinity intensity plots after applying Averaging and Gaussian filters (σ = 5) with different kernel sizes: (a) Vicinity intensity of 0.8 µm beads with Averaging filter. (b) Vicinity intensity of 0.8 µm beads with Gaussian filter. (c) Vicinity intensity of 1 µm beads with Averaging filter. (d) Vicinity intensity of 1 µm beads with Gaussian filter. (e) Vicinity intensity of 2 µm beads with Averaging filter. (f) Vicinity intensity of 2 µm beads with Gaussian filter. (g) Vicinity intensity of 8.3 µm beads with Averaging filter. (h) Vicinity intensity of 8.3 µm beads with Gaussian filter. Note: The numbers inside each bar picture represent 3x3x3 to 21x21x21 filters.

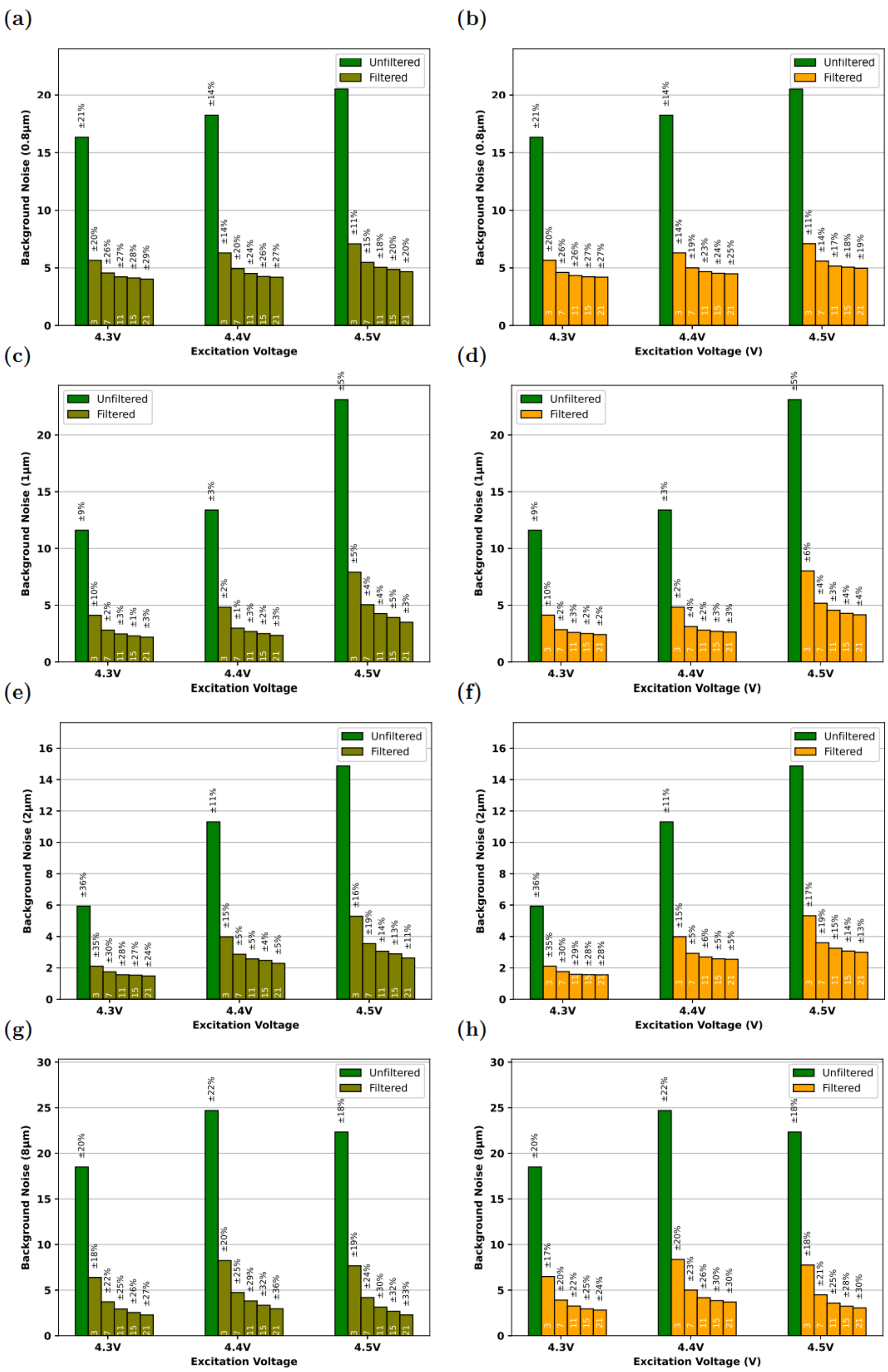


Fig. S10. Background noise intensity plots after applying Averaging and Gaussian filters (σ = 5) with different kernel sizes: (a) Background noise in 0.8 µm beads with Averaging filter. (b) Background noise in 0.8 µm beads with Gaussian filter. (c) Background noise in 1 µm beads with Averaging filter. (d) Background noise in 1 µm beads with Gaussian filter. (e) Background noise in 2 µm beads with Averaging filter. (f) Background noise in 2 µm beads with Gaussian filter. (g) Background noise in 8.3 µm beads with Averaging filter. (h) Background noise in 8.3 µm beads with Gaussian filter. Note: The numbers inside each bar picture represent 3x3x3 to 21x21x21 filters.

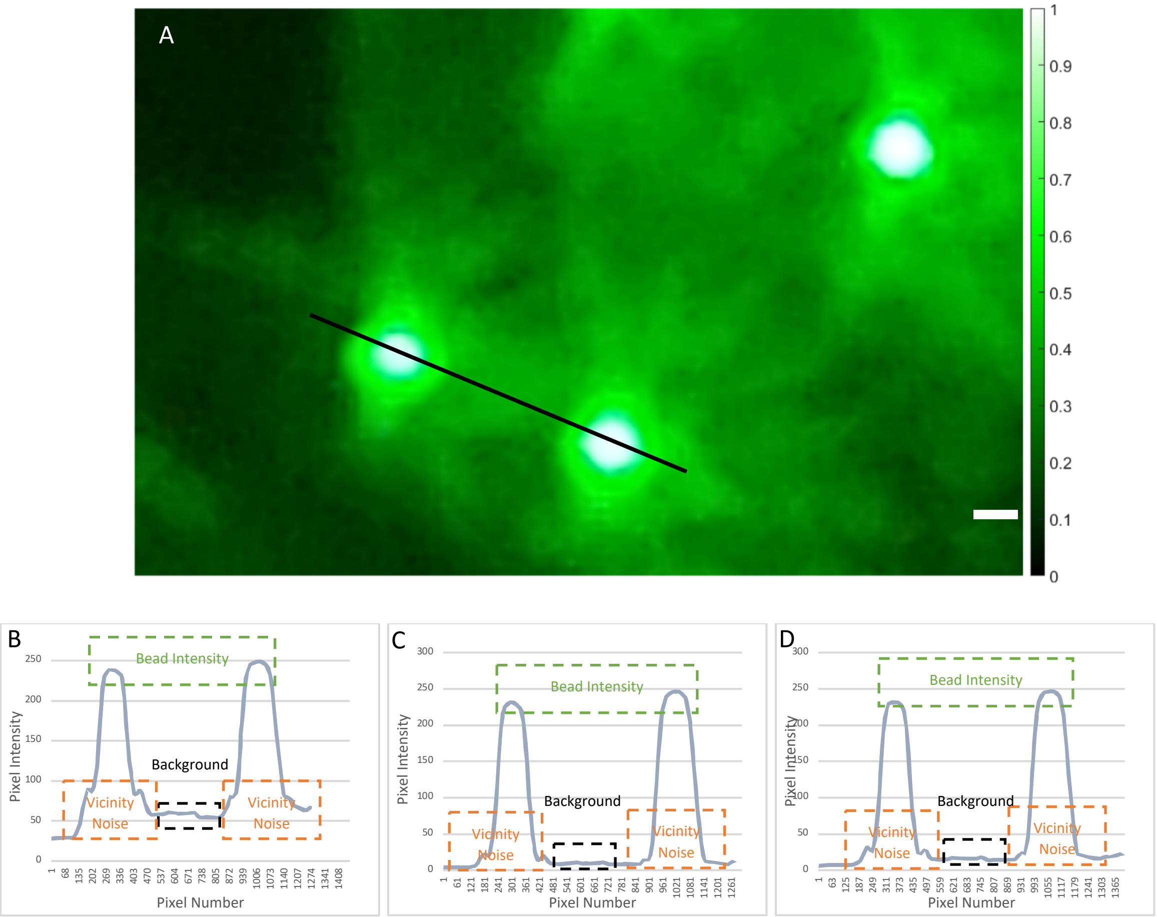


Fig.S11. (A) Image of the 8.3 µm beads used to create the intensity profile, the scale bar has been normalized to show pixels (0-255), where 1 corresponds to value 255. (B) Intensity of the 8.3 µm beads before applications of filters. (C) Intensity profile of the beads after applying an averaging filter. D) Intensity profile of the beads after applying the Gaussian filter. Scale bar 8µm.

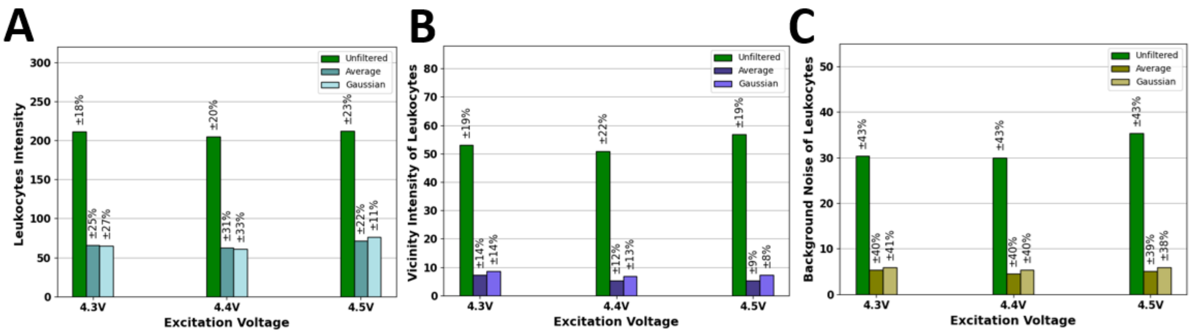


Fig. S12. (A) Mean leukocyte intensity before and after applying Average and Gaussian filter (σ = 5) of kernel size 21 x 21 x 21. (B) Mean leukocyte vicinity intensity before and after the application of average and Gaussian filter (σ = 5) of kernel size 21 x 21 x 21. (C) Background noise in leukocyte images before and after the application of average and Gaussian filter (σ = 5) of kernel size 21 x 21 x 21.

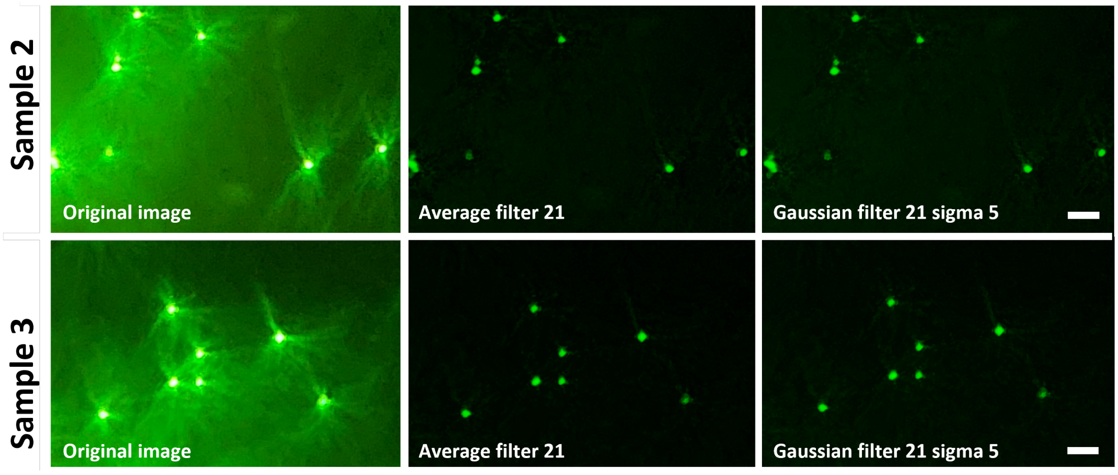


Fig. S13. Samples 2 and 3 of leukocytes imaged at 4.5V before and after the application of Averaging and Gaussian filters (σ = 5) of kernel size 21 x 21 x 21 (scale bar = 25 µm). Note: The numbers on each picture represent 21x21x21 filters.

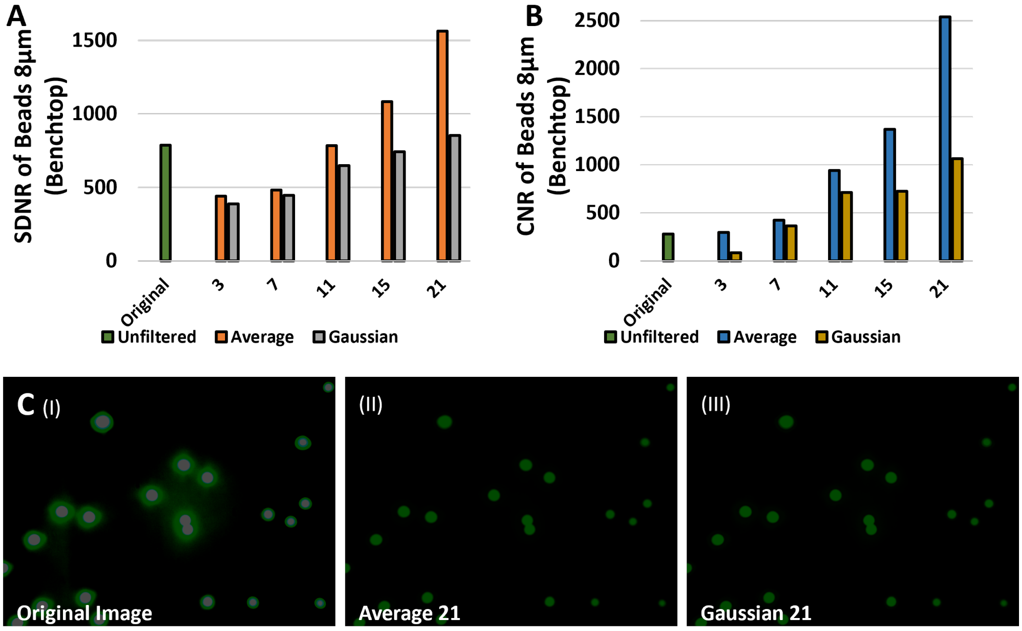


Fig. S14. (A) SDNR improvement in the image of 8.3 µm bead imaged using a benchtop microscope after application of averaging and Gaussian filters. (B) CNR improvement after application of averaging and Gaussian filters. (C) (I) Original unfiltered image of 8.3 µm bead. (II) Filtered image after applying an averaging filter of 21 x 21 x 21. (III) Filtered image after applying the Gaussian filter of 21 x 21 x 21 (scale bar = 25 µm). Note: The numbers on each picture represent 21x21x21 filters.

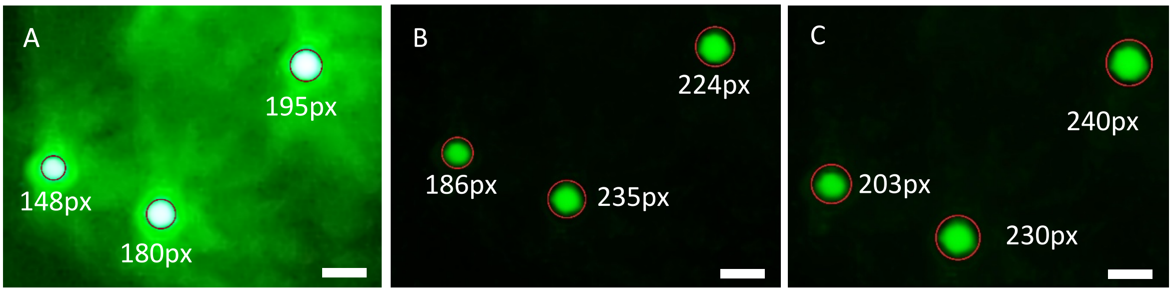


Fig. S15: A) Visual representation of the 8.3 µm beads before applications of filters. (scale bar 8 µm). B) The image results after applying the averaging filter and change in the bead size. C) The image results after applying the Gaussian filter and change in the bead size.

# Spatial Resolution Analysis

We performed the spatial resolution estimation before and after the application of the filters. The spatial resolution will affect the samples containing large numbers of beads with smaller sizes in close proximity. Therefore, we performed the spatial resolution analysis on the samples for 0.8µm beads. Fig. S11 shows the comparison between the figures. It was observed from Fig. S16(A) that before the application of the filters, the distance between the beads separated by the red line was 60 px (22µm), and it increased to 75px(28µm) after the application of both Averaging and Gaussian filters.

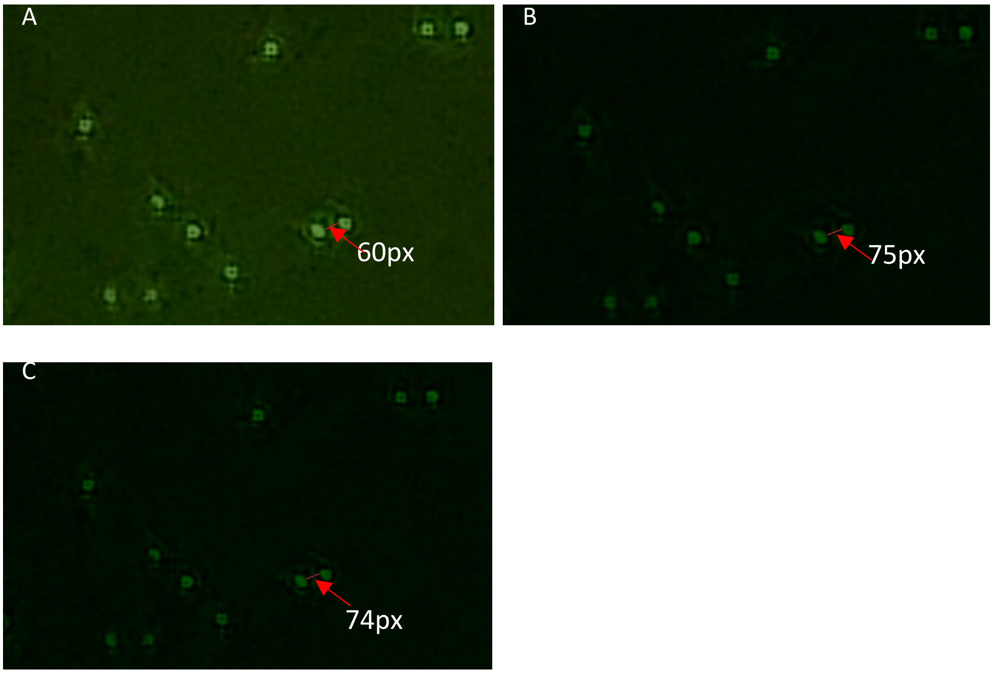


Fig. S16: A) Estimating the spatial resolution on 0.8µm beads image before filtering. B) Estimating the spatial resolution on 0.8µm beads image after Averaging filtering. C) Estimating the spatial resolution on 0.8µm beads image after Gaussian filtering.

# Supplementary Tables

Table S1: Comparing performance and efficacy of the current methods against the previously published methods

| | Method | Metric | Comment |
|---|---|---|---|
| **Breslauer et al. [1]** | Optical Hardware | - | The method relied on using a higher aperture of 0.85 NA and 60x magnification to achieve high resolution. |
| **Arpa et al. [2]** | Optical Hardware | - | Using a second smartphone display as the background illumination allows enhanced microscopic images to be captured |
| **Jiao et al. [3]** | Deep Learning (U-net for feature extraction, VGG for particle classification and counting) | - | Didn't report any enhancement quantifying metrics; rather, relied on the classification accuracy of particle counting models (80%) |
| **Hu et al. [4]** | Deep Learning (Custom network containing convolutional and residual blocks) | PSNR (29 dB) | Improved the PSNR of the image from 28 to 29 dB. Model trains a DL model, which compresses and decompresses the image, resulting in a denoised image. |
| **Chen et al. [5]** | Deep Learning (Compressed blind deconvolution and denoising) | PSNR (31dB) | Improved the PSNR from 26 to 31. The model trained on the deep convolutional networks generates the denoised image. |
| **Rivenson et al. [6]** | Deep learning (Convolutional neural networks) | Color Difference between benchtop and smartphone (4.3) | Trained a deep learning model to reconstruct the images and calculated the difference between output and benchtop image, improving the value from 17 to 4.3 |
| **Our Work** | Statistical (Averaging and Gaussian Filters) | SDNR (upto 35dB) | We applied the filters and then used our previously developed method to calculate the parameters and significantly improved the SDNR from 10DB to 35dB. |

Table S2: Optimal excitation voltage for the four different green fluorescent beads imaged using the presented smartphone fluorescence microscope (SFM).

| Bead size | Possible excitation voltages | Optimal imaging voltages |
|---|---|---|
| **8 µm** | 3.8 to 4.5V | 3.8V |
| **2 µm** | 4.1 to 4.5V | 4.1 to 4.3V |
| **1 µm** | 4.3 to 4.5V | 4.3 to 4.5V |
| **0.8 µm** | 4.3 to 4.5V | 4.3 to 4.5V |

Table S3: Quantitative feature comparison of the presented SFM design with respect to previously published designs by researchers.

NR: Not relevant

NP: Nano particles

| Reference | Optical Resolution | Optical Magnification | Correlation w.r.t benchtop instrument | Smallest feature imaged | Field of view | Imaging Performance Enhancement |
|---|---|---|---|---|---|---|
| [13] | NR | 0.14 | 0.99 | NR | NR | No |
| [14] | NR | 1.625 | 0.89 | 0.5 µm | 2 $mm^2$ | No |
| [15] | 0.98 µm | 2.6 | 0.99 | NR | 0.8 $mm^2$ | No |
| [16] | NR | 1 | 0.98 | Approx 7 µm | 14 $mm^2$ | No |
| [17] | 6.2 µm | 0.24 | 0.99 | Approx 7 µm | 80 $mm^2$ | No |
| [18] | 2 µm | NR | NR | 6 µm | 4 $mm^2$ | No |
| [19] | 2.76 µm | NR | NR | NR | 1.4 $mm^2$ | No |
| [20] | NR | NR | 0.97 | 5.35 µm | NR | No |
| [21] | 6.2 µm | 0.24 | 0.99 | 8 µm | 80 $mm^2$ | No |
| [22] | NR | NR | 1 | 16.2 µm | NR | No |
| **Presented Work** | **2.2 µm** | **2.16** | **0.99** | **800 nm (NPs)** | **3 $mm^2$** | **Yes** |

Table S4: Cost analysis of the SFM.

| Component | Catalog Number | Price($) | Date | Provider |
|---|---|---|---|---|
| **3.1 mm focal length lens** | 87-165 | 113.46 | 06/04/2025 | Edmund Optics |
| **Long pass emission filter filter with a cut-off value of 500 nm** | FF01-500/LP-23.3-D | 333.3 | 06/04/2025 | Semrock |
| **10 mm focal length lens** | 45-208 | 68.5 | 06/04/2025 | Edmund Optics |
| **Laser diode** | PLT5 450GB | 23.26 | 06/04/2025 | Mouser Electronics |
| **Excitation Filter** | ET470/40x | 365 | 06/04/2025 | Chroma |
| **3D printed device** | | 10 | | In house printing |
| | Total | $914 | | |

Table S5: Contrast and noise values for bead images estimated before and after the application of the Averaging filters of different kernel sizes.

| Bead size | Parameter | Unfiltered | Images filtered with the Averaging filter. | | | | |
|---|---|---|---|---|---|---|---|
| | | | 3x3x3 | 7x7x7 | 11x11x11 | 15x15x15 | 21x21x21 |
| 8 µm | Contrast | 3.29 | 3.21 | 7.58 | 14.31 | 14.31 | 18.55 |
| | Noise | 17.63 | 6.01 | 3.07 | 2.23 | 1.83 | 1.46 |
| | Contrast to noise ratio | 0.20 | 0.57 | 2.69 | 5.65 | 9.07 | 15.46 |
| 2 µm | Contrast | 4.81 | 4.61 | 6.76 | 7.81 | 8.45 | 8.73 |
| | Noise | 12.92 | 4.67 | 3.08 | 2.77 | 2.64 | 2.45 |
| | Contrast to noise ratio | 0.38 | 1.01 | 2.26 | 2.93 | 3.31 | 3.71 |
| 1 µm | Contrast | 2.03 | 1.93 | 2.75 | 3.07 | 3.26 | 3.32 |
| | Noise | 22.37 | 7.65 | 5.02 | 4.38 | 3.99 | 3.55 |
| | Contrast to noise ratio | 0.09 | 0.26 | 0.56 | 0.72 | 0.84 | 0.96 |
| 0.8 µm | Contrast | 2.61 | 2.49 | 3.22 | 3.44 | 3.49 | 3.42 |
| | Noise | 17.26 | 6.01 | 4.51 | 4.01 | 3.76 | 3.61 |
| | Contrast to noise ratio | 0.16 | 0.43 | 0.74 | 0.89 | 0.96 | 0.98 |

Table S6: Contrast and noise values for bead images estimated before and after the application of the Gaussian filters of different kernel sizes.

| Bead size | Parameter | Unfiltered | Images filtered with the Gaussian filter. | | | | |
|---|---|---|---|---|---|---|---|
| | | | 3x3x3 | 7x7x7 | 11x11x11 | 15x15x15 | 21x21x21 |
| 8 µm | Contrast | 3.29 | 3.16 | 7.06 | 9.62 | 11.11 | 12.12 |
| | Noise | 17.63 | 6.12 | 3.38 | 2.64 | 2.24 | 2.06 |
| | Contrast to noise ratio | 0.20 | 0.55 | 2.27 | 4.02 | 5.56 | 6.62 |
| 2 µm | Contrast | 4.81 | 4.59 | 6.51 | 7.34 | 7.74 | 7.90 |
| | Noise | 12.92 | 4.68 | 3.12 | 2.92 | 2.78 | 2.74 |
| | Contrast to noise ratio | 0.38 | 1.01 | 2.15 | 2.60 | 2.89 | 2.99 |
| 1 µm | Contrast | 2.03 | 1.91 | 2.65 | 2.96 | 7.74 | 7.90 |
| | Noise | 22.37 | 7.71 | 5.12 | 4.57 | 4.40 | 4.18 |
| | Contrast to noise ratio | 0.09 | 0.25 | 0.53 | 0.66 | 0.71 | 0.75 |
| 0.8 µm | Contrast | 2.61 | 2.48 | 3.13 | 3.34 | 3.37 | 3.37 |
| | Noise | 17.26 | 6.03 | 4.68 | 4.14 | 4.02 | 3.87 |
| | Contrast to noise ratio | 0.16 | 0.43 | 0.69 | 0.83 | 0.87 | 0.90 |

Table S7: Contrast and noise values estimated before and after the application of the Averaging and Gaussian filter of size 21x21x21 on leukocyte images.

| Parameter | Unfiltered | Image filtered with the Averaging filter of size 21x21x21 | Image filtered with the Gaussian filter of size 21x21x21 |
|---|---|---|---|
| Contrast | 2.73 | 12.62 | 9.59 |
| Noise | 35.41 | 5.01 | 5.90 |
| Contrast to noise ratio | 0.08 | 2.54 | 1.63 |

Table S8: Data retention in the green fluorescent bead images after the application of Averaging filters of multiple kernel sizes.

| Bead size | Excitation Voltage | Normal count | Bead count of the filtered images | | | | |
|---|---|---|---|---|---|---|---|
| | | | 3x3x3 | 7x7x7 | 11x11x11 | 15x15x15 | 21x21x21 |
| 8 µm | 4.3 | 17 | 17 | 17 | 17 | 17 | 17 |
| | 4.4 | 17 | 17 | 17 | 17 | 17 | 17 |
| | 4.5 | 18 | 17 | 17 | 17 | 17 | 17 |
| 2 µm | 4.3 | 41 | 40 | 40 | 41 | 40 | 40 |
| | 4.4 | 38 | 38 | 38 | 38 | 40 | 41 |
| | 4.5 | 39 | 38 | 38 | 38 | 39 | 39 |
| 1 µm | 4.3 | 279 | 291 | 292 | 298 | 294 | 291 |
| | 4.4 | 289 | 295 | 295 | 291 | 293 | 286 |
| | 4.5 | 287 | 292 | 300 | 295 | 292 | 289 |
| 0.8 µm | 4.3 | 145 | 145 | 142 | 145 | 144 | 142 |
| | 4.4 | 150 | 146 | 143 | 148 | 148 | 146 |
| | 4.5 | 150 | 144 | 147 | 153 | 151 | 146 |

Table S9: Data retention in the green fluorescent bead images after the application of Gaussian filters (σ = 1) of multiple kernel sizes.

| Bead size | Excitation Voltage | Normal count | Bead count of the filtered images | | | | |
|---|---|---|---|---|---|---|---|
| | | | 3x3x3 | 7x7x7 | 11x11x11 | 15x15x15 | 21x21x21 |
| 8 µm | 4.3 | 17 | 17 | 17 | 17 | 17 | 17 |
| | 4.4 | 18 | 18 | 18 | 18 | 18 | 18 |
| | 4.5 | 18 | 18 | 18 | 18 | 18 | 18 |
| 2 µm | 4.3 | 41 | 40 | 40 | 40 | 41 | 40 |
| | 4.4 | 38 | 38 | 38 | 38 | 36 | 39 |
| | 4.5 | 39 | 39 | 37 | 39 | 39 | 38 |
| 1 µm | 4.3 | 279 | 294 | 294 | 294 | 294 | 294 |

| | | | | | | | |
|---|---|---|---|---|---|---|---|
| | 4.4 | 289 | 289 | 288 | 297 | 297 | 297 |
| | 4.5 | 287 | 300 | 294 | 294 | 294 | 294 |
| 0.8 µm | 4.3 | 145 | 143 | 147 | 148 | 148 | 144 |
| | 4.4 | 150 | 148 | 144 | 145 | 145 | 145 |
| | 4.5 | 150 | 150 | 142 | 146 | 146 | 146 |

Table S10: Data retention in the green fluorescent bead images after the application of Gaussian filters (σ = 3) of multiple kernel sizes.

| Bead size | Excitation Voltage | Normal count | Bead count of the filtered images | | | | |
|---|---|---|---|---|---|---|---|
| | | | 3x3x3 | 7x7x7 | 11x11x11 | 15x15x15 | 21x21x21 |
| 8 µm | 4.3 | 17 | 17 | 17 | 17 | 17 | 17 |
| | 4.4 | 18 | 18 | 18 | 18 | 18 | 18 |
| | 4.5 | 18 | 18 | 18 | 17 | 17 | 17 |
| 2 µm | 4.3 | 41 | 40 | 40 | 40 | 40 | 40 |
| | 4.4 | 38 | 38 | 38 | 38 | 38 | 38 |
| | 4.5 | 39 | 38 | 38 | 39 | 39 | 38 |
| 1 µm | 4.3 | 279 | 298 | 292 | 291 | 291 | 289 |
| | 4.4 | 289 | 297 | 294 | 290 | 290 | 289 |
| | 4.5 | 287 | 299 | 287 | 295 | 287 | 286 |
| 0.8 µm | 4.3 | 145 | 144 | 142 | 152 | 138 | 150 |
| | 4.4 | 150 | 150 | 141 | 144 | 146 | 147 |
| | 4.5 | 150 | 145 | 142 | 151 | 148 | 149 |

Table S11: Data retention in the green fluorescent bead images after the application of Gaussian filters (σ = 5) of multiple kernel sizes.

| Bead size | Excitation Voltage | Normal count | Bead count of the filtered images | | | | |
|---|---|---|---|---|---|---|---|
| | | | 3x3x3 | 7x7x7 | 11x11x11 | 15x15x15 | 21x21x21 |
| 8 µm | 4.3 | 17 | 17 | 17 | 17 | 17 | 17 |
| | 4.4 | 18 | 18 | 18 | 18 | 18 | 18 |
| | 4.5 | 18 | 18 | 18 | 17 | 17 | 17 |
| 2 µm | 4.3 | 41 | 40 | 40 | 40 | 40 | 40 |
| | 4.4 | 38 | 38 | 38 | 38 | 38 | 39 |
| | 4.5 | 39 | 42 | 38 | 38 | 37 | 39 |
| 1 µm | 4.3 | 279 | 292 | 292 | 283 | 298 | 298 |
| | 4.4 | 289 | 296 | 296 | 292 | 292 | 288 |
| | 4.5 | 287 | 296 | 307 | 296 | 295 | 306 |
| 0.8 µm | 4.3 | 145 | 145 | 149 | 147 | 143 | 145 |
| | 4.4 | 150 | 147 | 149 | 148 | 147 | 151 |
| | 4.5 | 150 | 143 | 147 | 152 | 151 | 159 |
